# Unraveling and leveraging in situ surface amorphization for enhanced hydrogen evolution reaction in alkaline media

**DOI:** 10.1038/s41467-023-42221-6

**Published:** 2023-10-13

**Authors:** Qiang Fu, Lok Wing Wong, Fangyuan Zheng, Xiaodong Zheng, Chi Shing Tsang, Ka Hei Lai, Wenqian Shen, Thuc Hue Ly, Qingming Deng, Jiong Zhao

**Affiliations:** 1 Department of Applied Physics, The Hong Kong Polytechnic University, Kowloon, China; 2 Department of Chemistry and Center of Super-Diamond & Advanced Films (COSDAF), City University of Hong Kong, Kowloon, China; 3 Department of Chemistry and State Key Laboratory of Marine Pollution, City University of Hong Kong, Hong Kong, China; 4 City University of Hong Kong Shenzhen Research Institute, Shenzhen, China; 5 Phyics Department and Jiangsu Key Laboratory for Chemistry of Low-Demensional Materials, Huaiyin Normal University, Huaian, China; 6 The Hong Kong Polytechnic University Shenzhen Research Institute, Shenzhen, China

**Keywords:** Two-dimensional materials, Electrocatalysis

## Abstract

Surface amorphization provides electrocatalysts with more active sites and flexibility. However, there is still a lack of experimental observations and mechanistic explanations for the in situ amorphization process and its crucial role. Herein, we propose the concept that by in situ reconstructed amorphous surface, metal phosphorus trichalcogenides could intrinsically offer better catalytic performance for the alkaline hydrogen production. Trace Ru (0.81 wt.%) is doped into NiPS_3_ nanosheets for alkaline hydrogen production. Using in situ electrochemical transmission electron microscopy technique, we confirmed the amorphization process occurred on the edges of NiPS_3_ is critical for achieving superior activity. Comprehensive characterizations and theoretical calculations reveal Ru primarily stabilized at edges of NiPS_3_ through in situ formed amorphous layer containing bridging S_2_^2−^ species, which can effectively reduce the reaction energy barrier. This work emphasizes the critical role of in situ formed active layer and suggests its potential for optimizing catalytic activities of electrocatalysts.

## Abstract (web summary)

Surface amorphization generally provides electrocatalysts with more active sites and flexibility. Here it is employed in-situ liquid TEM to observe the surface reconstruction on Ru-NiPS3 nanosheets, confirming that the amorphization on the edges of NiPS3 is critical for achieving superior activity.

## Introduction

Electrochemical water splitting is a significant hydrogen production technique and involves a complex surface chemical reaction^1,2^. The catalytic activity is highly dependent on the surface structure of the catalytic materials, particularly in the case of alkaline water electrolysis (AWE)^3–5^. For the alkaline HER process, surface reconstruction is a frequently observed phenomenon and usually leads to the formation of an amorphous layer outside the catalysts^6,7^. However, the fundamental understanding of the surface amorphization process and the role of the resulting amorphous layer on catalysts is still deficient. Additionally, how to effectively leverage the inevitable amorphous layer to further improve the catalytic activity of electrocatalytic material remains a challenging task^8^. Hence, it is imperative to identify a proper material platform to comprehensively investigate the impact of the amorphous layer on the catalytic activity.

Recently, two-dimensional (2D) metal phosphorus trichalcogenides (MPTs) have garnered increasing attention as catalysts for the hydrogen evolution reaction (HER)^9–13^. Compared with other reported transition metal-based electrocatalysts, 2D MPTs demonstrate unique crystal structures with a metal layer encapsulated by both chalcogens and phosphorus atoms, as well as abundant [P_*2*_S_*6*_]^4–^ functional groups. These features provide high specific surface area, tunable charge states, and appropriate band structures^12,14–16^. Due to the semiconductor nature and inert basal plane of MPTs, the catalytic active sites are mainly concentrated at the edge positions^13^. Therefore, using 2D MPTs as a research subject can effectively mitigate the influence of extraneous factors, enabling more precise investigations into the effects of the amorphous layer on catalytic sites and the overall activity of the material.

Herein, we introduce an edge optimization strategy to enhance the catalytic activity of MPTs. We leverage the inevitable surface reconstruction that occurs during the alkaline HER process to stabilize dopants (Ru) in the in situ formed amorphous layer, thereby enhancing the adsorption ability for intermediates, and increasing the number of active sites. The modified Ru-NiPS_3_ nanosheets (NSs) demonstrated an overpotential (*η*_*10*_) of 58 mV to reach the current density of 10 mA cm^−2^ and a high exchange current density of 1180 μA cm^−2^, which is comparable to the commercial Pt/C catalyst. Comprehensive characterizations and density functional theory (DFT) calculations revealed that the Ru atoms were trapped by the in situ formed bridging S_2_^2−^ species in the amorphous layer. This reduces the leaching of Ru dopant and modifies the electronic structure around the active sites. These findings confirm the benefits of in situ surface amorphization in transition metal-based electrocatalysts and provide an alternative avenue for designing highly efficient catalysts for electrochemical applications.

## Results and discussion

### Synthesis and structural characterization

Ru-NiPS_3_ NSs electrode was prepared through a three-step procedure (as shown in Supplementary Fig. 1). The obtained Ni precursor was first characterized with X-ray diffraction (PXRD) and scanning electron microscopy (SEM) image (Supplementary Figs. 2, 3), which demonstrated NSs morphology. For comparison, single crystal NiPS_3_ as a control sample was also prepared with a CVT method (details can be found in the experimental section in Supporting Information).

**Fig. 1:**
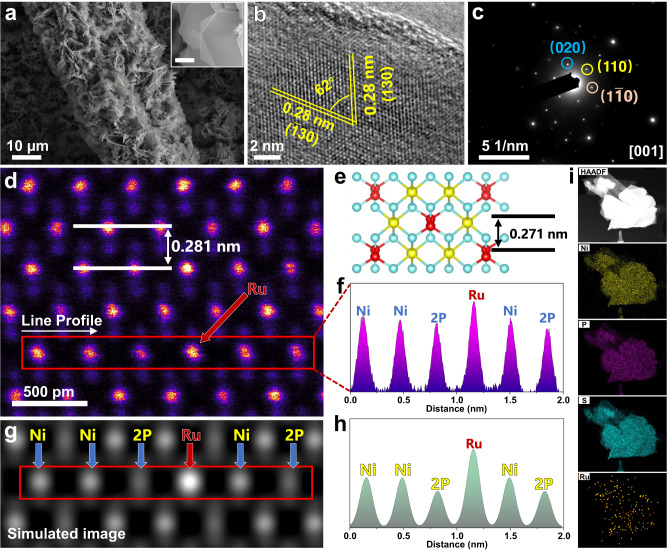
Structural characterizations of Ru-NiPS_3_ NSs. **a** SEM image of the as-prepared Ru-NiPS_3_ NSs. **b**, **c** HRTEM image and the corresponding SAED image along the [001] zone axis. **d** Atomic-level HAADF-STEM image of an ultrathin NiPS_3_ nanosheet. **e** Crystal structure of NiPS_3_ along [001] zone axis. **f** Line intensity profile obtained from the selected area in (**d**). (**g**) and (**h**) are the simulated HAADF-STEM image with Ru doped into the NiPS_3_ lattice and the corresponding line intensity profile which is similar to the experimental one. **i** HDDF-STEM image corresponding EDS mapping of Ru-NiPS_3_ nanosheet (scale bar 500 nm).

We thoroughly examined the morphology and crystal structure of our prepared samples. The SEM images demonstrated that the Ru-NiPS_3_ nanosheets had grown uniformly in a hexagonal shape on the surface of carbon cloth with an average thickness of about 150 nm (Fig. 1a and Supplementary Figs. 4, 5). We also characterized the morphology and crystal structure of NiPS_3_ NSs without Ru doping for comparison and found that they were similar in shape to the Ru-NiPS_3_ nanosheets. (Supplementary Figs. 6, 7a). To confirm the quality of our NiPS_3_ nanosheets, we further analyzed their energy-dispersive X-ray spectroscopy spectra, which showed a stoichiometric ratio of Ni:P:S = 1:1:3 (Supplementary Fig. 7b).

Samples that were immersed in RuCl_3_ solution for varying durations demonstrated comparable morphology to the Ru-NiPS3 nanosheets described earlier (Supplementary Figs. 8–11). The high-resolution transmission electron microscopy (HRTEM) image of Ru-NiPS_3_ NSs showed two different interplanar distances of 0.286 nm and 0.280 nm with a dihedral angle of 62°, corresponding to the (130) and ($\bar{1}$30) planes (Fig. 1b)^17^. The corresponding selected area electron diffraction (SAED) pattern was indexed to the monoclinic crystal structure along the [001] zone axis (Fig. 1c), which closely matched the theoretical NiPS_3_ crystal. To determine the precise distribution of Ru atoms within the nanosheets, aberration-corrected high-angle annular dark-field scanning transmission electron microscopy (AC HAADF-STEM) imaging characterization was conducted (Fig. 1d). The AC HAADF-STEM image was taken along the [001] zone axis, with an adjacent lattice fringe of 0.281 nm, which matched well with the theoretical NiPS_3_ crystal (Fig. 1e). By analyzing the intensity profile of the nanosheets, it was confirmed that the Ru atoms were doped into the NiPS_3_ lattice site, occupying the same position as the Ni atoms (Fig. 1f), which is consistent with the simulated HAADF image and the corresponding intensity profile (Fig. 1g, h).

To further verify the position of the Ru atoms within the nanosheets, we also obtained the AC HAADF-STEM image along the [013] zone axis, which confirmed that the Ru atoms had replaced the Ni atoms during the ion exchange process (Supplementary Fig. 12). At the same time, STEM-EDS mapping showed clearly that Ni, P, S, and Ru elements were uniformly distributed within the nanosheet (Fig. 1i). Finally, we used inductively coupled plasma-optical emission spectroscopy (ICP-OES) analysis to confirm the amount of Ru in the nanosheets and it revealed that the Ru content was 0.81 wt.% (Supplementary Table 1). The ICP-OES results of other samples treated with different dipping durations were demonstrated in (Supplementary Table 2), which indicated that the maximum loading of Ru species by ion exchange methods is about 0.8 wt.%.

**Fig. 2:**
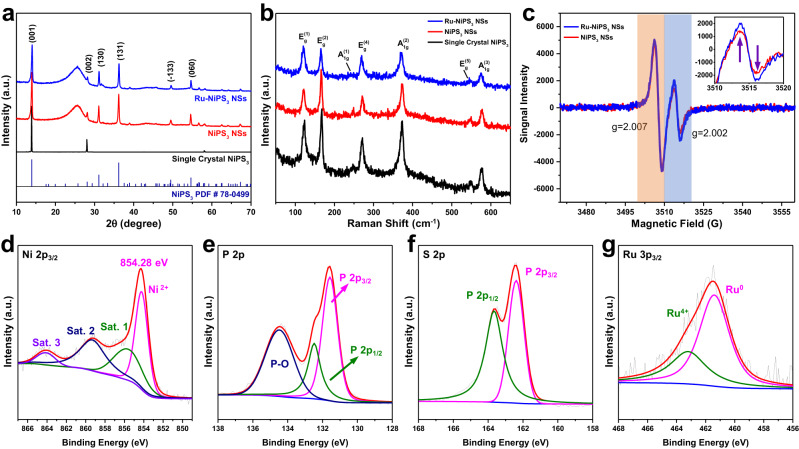
Compositional and electronic structure of as-prepared electrocatalysts. **a** PXRD patterns of Single crystal NiPS_3_ powder, pure NiPS_3_ NSs, and Ru-NiPS_3_ NSs. **b** Raman spectra of the samples as mentioned above. **c** EPR results of NiPS_3_ and Ru-NPS_3_. XPS spectra of (**d**) Ni 2p_3/2_, (**e**) P 2p, (f) S 2p, and (**g**) Ru 3p.

To gain further insights into the structure and chemical states of the prepared samples, we used several other analytical techniques, including powder X-ray diffraction (PXRD), Raman spectroscopy, and X-ray photoelectron spectroscopy (XPS). As shown in Fig. 2a, the single crystalline NiPS_3_ showed strong diffraction peak intensity of (001), (002) and (004) which belonged to NiPS_3_ (PDF #78-0499, space group: *C2/m*, and unit cell parameters: *a* = 5.812 Å, *b* = 10.070 Å, and *c* = 6.632 Å), indicating the high quality of our prepared sample. Meanwhile, the PXRD patterns for both the NiPS_3_ nanosheets and the Ru-doped NiPS_3_ nanosheets revealed their polycrystalline structure and the varying immersing durations during sample preparation will not change the crystal structure of NiPS_3_ (Fig. 2a and Supplementary Fig. 13).

The Raman spectra of different samples demonstrated four in-plane E_g_ and three out-of-plane A_1g_ vibrational modes within the range of 100–600 cm^−1^. The peak below 150 cm^−1^ was attributed to the Ni^2+^ metal ion vibrations in NiPS_3_, while other peaks below 600 cm^−1^ were associated with the vibrational modes of the PS_3_ group and P-P bond (Fig. 2b and Supplementary Fig. 14)^18^. The electron paramagnetic resonance (EPR) spectra of NiPS_3_ and Ru-NiPS_3_ were performed to study the concentration of vacancies. Two different symmetric EPR peaks were detected at *g* = 2.002 and 2.007, which were attributed to the presence of P vacancies (P-V)^19^ and S vacancies (S-V)^18^, respectively. The intensity of the P-V peak was similar in both samples, as revealed by the EPR spectra. However, the Ru-doped nanosheets exhibited a slightly higher intensity of the S-V peak compared to the pristine one, suggesting a higher concentration of S-V in Ru-NiPS_3_ NSs (Fig. 2c). Usually, a higher concentration of S vacancy would provide more active sites and result in better HER activity^20^. As observed in the AC HAADF-STEM image, Ru atoms would replace Ni atoms after ion exchange. Considering the crystal structure characteristics (a Ni/P atom would coordinate with 6S atoms), the increment of S-V further indicated Ru would occupy Ni sites when doped into the lattice.

X-ray photoelectron spectroscopy (XPS) was performed to understand the surface chemical state of the Ru-NiPS_3_ electrode (survey spectrum was demonstrated in Supplementary Fig. 15). The Ni 2p_3/2_ spectrum was deconvoluted into four peaks, indicating the presence of core levels of Ni^2+^ (854.3 eV) and three corresponding satellite peaks located at 855.9 eV, 859.4 eV, and 864.2 eV, respectively^21,22^. The formation of NiPS_3_ was further supported by the observation of three clear satellite peaks, which indicate the hybridization of Ni^2+^ level and PS_3_ ligand orbitals (Fig. 2d)^13^. The P 2p spectrum exhibited two distinct peaks at 131.6 eV and 132.5 eV, corresponding to P 2p_3/2_ and P 2p_1/2_, respectively, providing evidence for the formation of covalent P–S bonds within the PS_3_ units. Furthermore, a broad peak at 134.5 eV was observed, likely arising from surface oxidation during the synthesis of transition metal-based phosphides (Fig. 2e)^23^. The S 2p spectrum also demonstrated two peaks at 162.4 eV and 163.7 eV, which belonged to the S 2p_3/2_ and S 2p_1/2_ spin–orbit peaks, respectively (Fig. 2f)^24^. Ru 3p_3/2_ spectrum exhibited two deconvoluted peaks at 461.4 eV and 463.2 eV, which could be ascribed to Ru^0^ and Ru^4+^ ^25^. The XPS spectra for undoped NiPS_3_ NSs electrodes were also demonstrated for better comparisons. The XPS spectrum of Ni 2p_3/2_ and P 2p demonstrated similar deconvoluted peaks as the Ru-NiPS_3_ sample without obvious peak shift (Supplementary Fig. 16a, b). However, the binding energy for S 2p_3/2_ (162.2 eV) and S 2p_1/2_ (163.4 eV) were lower than the Ru-NiPS_3_ NSs, indicating the formation of Ru-S bond, and more electrons accumulated around Ru atoms due to its high electronegativity (Ru (2.2) and Ni (1.9)) (Supplementary Fig. 16c)^26^.

### Electrocatalytic performances of Ru-NiPS_3_ NSs

**Fig. 3:**
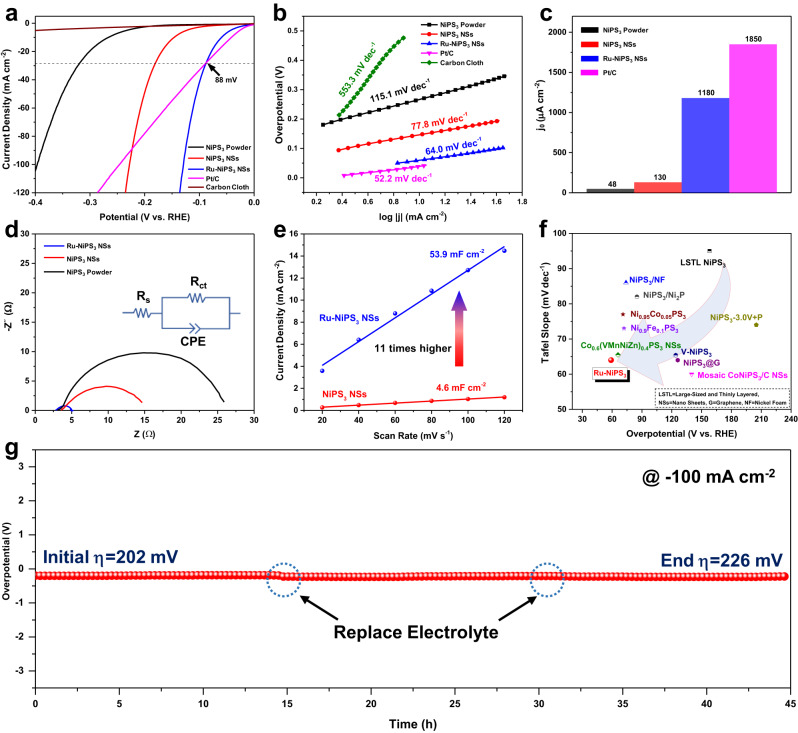
HER performances of different samples in 1 M KOH (pH = 14). **a** HER polarization curves for carbon cloth, NiPS_3_ powder, NiPS_3_ NSs, Ru-NiPS_3_ NSs, and Pt/C measured in 1 M KOH with a scan rate of 2 mV s^−1^. The mass loading of the electrocatalysts is ~1.5 mg cm^−2^, and the solution resistance is ~3.0 Ω. **b** Tafel slope of different catalysts obtained from the polarization curves in (**a**). **c** Exchange current density of different samples extrapolated linearly from the Tafel slope in (**b**). **d** Nyquist plots of NiPS_3_ powder, NiPS_3_ NSs, and Ru-NiPS_3_ NS (inset shows the equivalent circuit diagram). (**e**) Scan rate dependence of the average capacitive currents for NiPS_3_ NSs and Ru-NiPS_3_ NSs. **f** Comparison of the overpotential and Tafel slope among our catalyst and other reported NiPS3-based electrocatalysts for alkaline HER. **g** Chronoamperometry curve test for Ru-NiPS_3_ NSs at a fixed current density of −100 mA cm^−2^.

We investigated the hydrogen evolution reaction (HER) activity of Ru-doped NiPS_3_ in 1 M KOH electrolyte. The electrode that had been dipped in RuCl_3_ solution for 16 h demonstrated the most effective catalytic activity and was chosen as the representative sample (Supplementary Figs. 17, 18). As shown in Fig. 3a, Ru-NiPS_3_ NSs exhibited a relatively low *η*_*10*_ of 59 mV to achieve a current density of 10 mA cm^−2^, which is significantly lower than the undoped NiPS_3_ NSs (*η*_*10*_ = 146 mV). Moreover, the HER activity of Ru-NiPS_3_ NSs was comparable to the commercial Pt/C electrocatalysts (*η*_*10*_ = 41mV) and surpassed the latter when the overpotential reached 88 mV. Due to the low specific area and less exposed active sites, NiPS_3_ powder showed the lowest HER performance with an *η*_*10*_ of 266 mV to achieve current densities of 10 mA cm^−2^. Tafel plots derived from the polarization curves showed that Ru-NiPS_3_ NSs have the lowest Tafel slope of 64.0 mV dec^−1^, which is much lower than the NiPS_3_ NSs (77.8 mV dec^−1^) and NiPS_3_ powder (115.1 mV dec^−1^), indicating a faster HER kinetics process through Volmer–Heyrovsky mechanism (Fig. 3b)^27,28^. The exchange current density for each sample was obtained using the Tafel plot extrapolation method. Ru-NiPS_3_ NSs exhibited a current density of 1180 μA cm^−2^, which is much larger than NiPS_3_ NSs (130 μA cm^−2^) and NiPS_3_ powder (48 μA cm^−2^), further indicating an enhanced intrinsic HER activity for Ru-NiPS_3_ NSs (Fig. 3c)^2^.

The HER kinetics for each sample was then investigated by electrochemical impedance spectroscopy (EIS). The Ru-NiPS_3_ NSs demonstrated a lower charge transfer resistance (*R*_ct_) compared to NiPS_3_ powder and NiPS_3_ NSs (Fig. 3d and Supplementary Table 3), suggesting that Ru-NiPS_3_ NSs may have faster charge transfer and ultimately improved HER activities. Electrochemical double-layer capacitance (*C*_dl_) was then utilized to estimate the electrochemical surface area (ECSA) and the concentration of catalytic active sites in each sample (Supplementary Figs. 19, 20). As shown in Fig. 3e and Fig. 18d, the value of *C*_dl_ would gradually increase with increasing dipping duration. The Ru-NiPS_3_ NSs demonstrated a *C*_dl_ of 53.9 mF cm^−2^, which is 11 times higher than that of the undoped NiPS_3_ NSs (4.6 mF cm^−2^), indicating the presence of a greater number of active sites available for HER.

Notably, the prepared Ru-NiPS_3_ NSs demonstrated superior HER performance compared to most of the previously reported NiPS_3_-based electrocatalysts (a summary of the data is presented in Fig. 3f and the data is extracted from Supplementary Table 4). The stability of the Ru-doped NiPS3 NSs electrode was assessed using the chronopotentiometry technique at a constant current density of 100 mA cm^−2^. Impressively, the electrode demonstrated only a minor 24 mV increase in overpotential after continuous testing for 45 h, indicating its remarkable durability (Fig. 3g).

Temperature-dependent kinetic analysis was conducted to further study the origin of the enhanced catalytic activities^29^. LSV curves for Ru-NiPS_3_ NSs and NiPS_3_ NSs were obtained under different temperatures (from 25 °C to 65 °C) for the calculation of the activation energy (*E*_app_) and pre-exponential factor (*A*_app_), which are two essential parameters to evaluate the catalytic mechanism. As expected, the HER performances of both Ru-NiPS_3_ NSs and NiPS_3_ NSs were improved with increasing temperature (Supplementary Fig. 21). *E*_app_ and *A*_app_ were then calculated according to the Arrhenius equation by fitting the Arrhenius curve (Supplementary Fig. 22). It was found that the maximum value of *E*_app_ for NiPS_3_ NSs appeared around its onset potential, while the maximum value for Ru-NiPS_3_ was at a relatively high overpotential. For the HER process in alkaline electrolytes, the rate-determining step (RDS) was the Volmer step, i.e., the water dissociation step due to the low concentration of H^+^ in alkaline media, which consisted of the situation for NiPS_3_ NSs^30^. However, the value of *E*_app_ for Ru-NiPS_3_ at the onset potential was much lower than its maximum at around 120 mV, indicating the RDS was no longer the Volmer step, and the desorption of H on the catalyst surface may be the limiting step for Ru-NiPS_3_ (Supplementary Fig. 23a)^31^. The value of *A*_app_ for Ru-NiPS_3_ is higher than that of NiPS_3_ NSs, suggesting a larger number of active sites participating in the HER (Supplementary Fig. 23b)^32–34^. The comprehensive electrochemical characterizations demonstrated that the incorporation of Ru into NiPS_3_ NSs substantially modifies the reaction path, leading to a reduction in the kinetic barrier for the formation of intermediates during the Volmer step in alkaline solutions^5^.

### Observation and characterization of surface reconstruction process

**Fig. 4:**
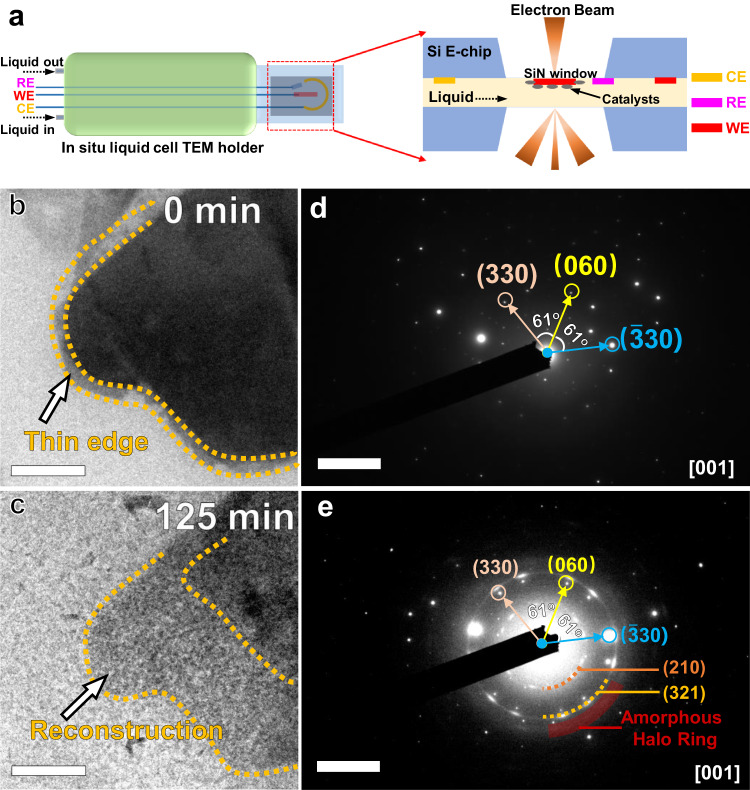
The structural evolution of Ru-NiPS_3_ NSs during the HER process. **a** Schematic illustration of the in situ electrochemical liquid cell TEM holder and the liquid cell. In situ liquid TEM image (scale bar: 0.2 μm) of Ru-NiPS_3_ NSs (**b**) before and (**c**) after chronopotentiometry test. **d**, **e** demonstrated the corresponding SAED patterns (scale bar: 5 1/nm) for (**b**) and (**c**), respectively. The in situ TEM image provided clear evidence of a reconstruction process occurring at the edges of the NSs during the HER process. Additionally, the corresponding SAED pattern confirmed that a portion of the NSs underwent a transformation into a polycrystalline and amorphous state.

In situ liquid electrochemical TEM technique was conducted to gain insights into the structural evolution of Ru-NiPS_3_ NSs during the alkaline HER process (Fig. 4a demonstrated the structure of the in situ electrochemical liquid cell TEM holder and the liquid cell). To minimize interference from other factors in the analysis, we first ruled out two potential sources of disturbance: corrosion of the sample by the alkaline solution, and interference from the electron beam on the sample. The Ru-NiPS_3_ NSs electrode was first immersed into 1 M KOH for 24 h to show the influence of the alkaline electrolyte. As demonstrated in Supplementary Fig. 24a, the morphology demonstrated negligible variation after the immersing process, which is also proved by the corresponding XRD pattern (Supplementary Fig. 24b) and Raman spectra (Supplementary Fig. 24c). To rule out the influence of the electron beam in the TEM, the assembled liquid in situ electrochemical TEM cell was placed into the TEM, and upon in situ irradiation, no significant changes to the sample were observed (Supplementary Movie 1). The above result demonstrated that both alkaline solution and electron beam in TEM have little influence on the sample. The in situ liquid electrochemical TEM measurement was conducted under a constant current of -5 nA vs. Pt (Supplementary Fig. 25). After continuously subjecting the Ru-NiPS_3_ NSs to a 2-h chronopotentiometry test, a significant reconstruction was observed at the edge position of the NSs (Fig. 4b, c). The corresponding SAED patterns clearly showed that while most of the NSs remained unchanged after the chronopotentiometry test (with similar diffraction spots as in Fig. 4d), a portion of the nanosheet underwent a transformation into a polycrystalline or amorphous state during the reconstruction process (as indicated by the presence of faint polycrystalline rings and amorphous halo ring in Fig. 4e). Detailed TEM images taken under different reaction time conditions show that the nanosheets underwent a gradual amorphization process, particularly at the edges (Supplementary Figs. 26 and 27). It is also found that the amorphization process would be more obvious at the thinner edges, which contribute to the formation of the functional amorphous layer for electrochemical reaction (Supplementary Fig. 28 and Supplementary Movie 2). According to some previous work, the amorphous layer can also protect the inner part of the nanosheet from over-etching, and effectively enhance the stability. Ex situ HRTEM images of Ru-NiPS_3_ after stability tests for different reaction duration (from 1 to 16 h) are demonstrated in Supplementary Fig. 29, which showed that with the reaction time increases, the thickness of the in situ formed amorphous layer gradually increases, eventually reaching a roughly stable thickness (~7.5 nm for 16 h). According to some previous work, the amorphous layer can also protect the inner part of the nanosheet from over-etching, and effectively enhance the stability, which is proved by the in situ liquid TEM, which demonstrated that after the amorphization process is complete, the morphology and edges of the material undergo minimal observable changes (Supplementary Movie 3).

**Fig. 5:**
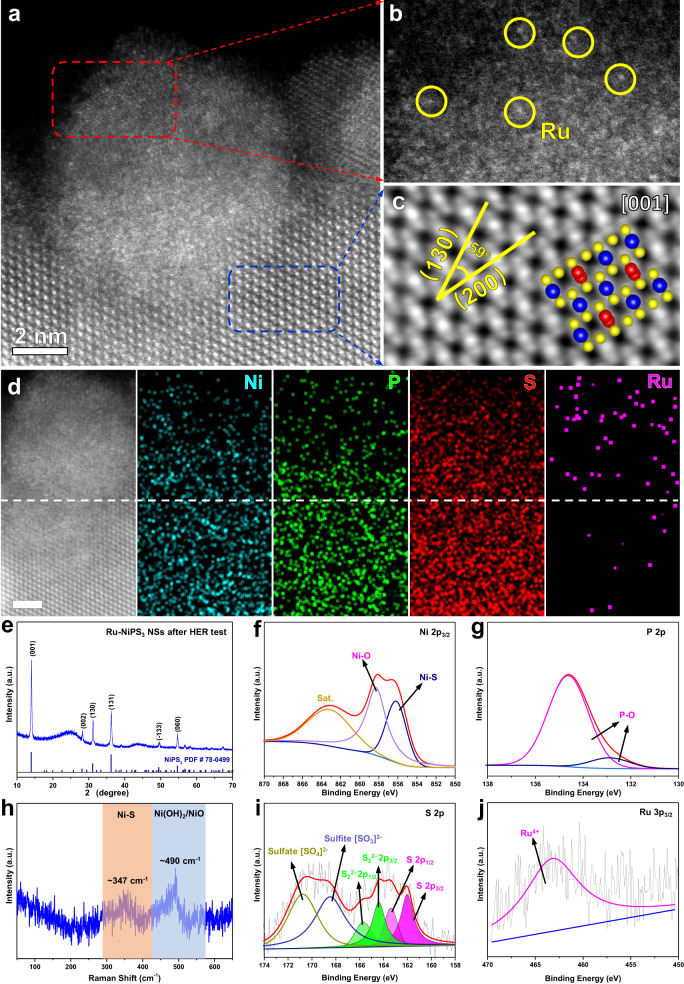
Characterizations of Ru-NiPS_3_ NSs after stability test. **a** HAADF-STEM image of Ru-NiPS_3_ after long-term stability HER test in 1 M KOH. **b**, **c** the enlarged part of amorphous part and inner part of Ru-NiPS_3_ from (**a**). **d** EDS mapping of Ni, P, S, and Ru element (scale bar: 2 nm). **e** PXRD image of Ru-NiPS_3_ NSs after HER test. **f** Raman spectrum of Ru-NiPS_3_ NSs after HER test. **g**–**j** XPS spectra for Ni 2p_3/2_, P 2p, S 2p, and Ru 3p_3/2_ of Ru-NiPS_3_ NSs after HER test.

Additional characterizations of the morphology and electronic structure of Ru-NiPS_3_ were conducted after the HER stability test, which were consistent with the in situ TEM characterizations. Compared with the pristine Ru-NiPS_3_ NSs, the edges of the NSs were partially transformed into an amorphous structure after the HER stability test (Fig. 5a, b), while the inner part still maintains the crystalline structure (Fig. 5c, and Supplementary Fig. 30). According to the HAADF-STEM image (Fig. 5b), Ru atoms were mainly distributed at the edge of the NSs. Furthermore, HAADF-STEM EDS element mapping revealed the uniform distribution of Ni, P, and S, while Ru atoms were found primarily in the amorphous region, forming a Ru-enriched shell (Fig. 5d). PXRD was used to characterize the crystal structure of the Ru-NiPS_3_ electrode after the HER stability test, which showed no significant change compared to the pristine sample. This finding suggests that the central part of the NSs remained as NiPS_3_, which would contribute to the stability of the NSs (Fig. 5e).

To further study the surface chemical state of the Ru-NiPS_3_ NSs after the HER stability test, Raman and XPS were employed. Raman spectra revealed only two broad peaks at approximately 347 cm^−1^ and 490 cm^−1^, which were attributed to Ni-S bonds and Ni oxides, respectively^35–37^, indicating the surface reconstruction of NiPS_3_ NSs (Fig. 5f). The Ni 2p_3/2_ spectra could be deconvoluted into three peaks. The peak located at 858.1 eV and 857.6 eV is attributed to the Ni^2+^ in Ni-S species and Ni-O bond, respectively, which is consistent with the Raman result (Fig. 5g)^38,39^. Different from the pristine Ru-NiPS_3_ NSs, only the peaks belonging to P–O bond were detected after HER test, which is inevitable during the alkaline HER process (Fig. 5h)^40,41^. The S 2p spectra were deconvoluted into two spin-orbit doublets, which correspond to the terminal S^2–^ (~162.1 eV and 162.9 eV) and bridging S_2_^2−^ (~163.9 eV and 164.6 eV)^42–45^. Another broad peak at ~169.6 eV was deconvoluted into two different peaks which belong to the sulfate species [SO_4_]^2-^ (at ~170.8 eV) and sulfite species [SO_3_]^2-^ (at ~168.5 eV) (Fig. 5i)^46^. In contrast to the pristine Ru-NiPS_3_, the Ru 3p_3/2_ spectra after the HER stability test exhibited only a single broad peak at around 463.1 eV, which was assigned to the Ru^4+^ species. Further ICP-OES measurements confirmed that the Ru species gradually dissolved into the electrolyte, and the remaining Ru^4+^ species served as the active species for the HER process (Supplementary Fig. 31). This observation suggests that the Ru^0^ or Ru cluster was unstable during the HER test, and only Ru^4+^ species remained in the NiPS_3_ NSs (Fig. 5j)^47,48^.

To further support the crucial role of Ru-enriched edges, the structure of undoped NiPS_3_ NSs was also characterized. Supplementary Figs. 32 and 33 demonstrate the presence of amorphous layer in the NiPS_3_ NSs after the HER stability test. However, the HER activity of NiPS_3_ was significantly lower than that of Ru-doped NSs, indicating the importance of Ru in the amorphous layer of Ru-NiPS_3_ NSs.

### Understanding the role of surface amorphous layer

DFT calculations were conducted to elucidate the origin of the enhanced HER performance for Ru-NiPS_3_ NSs. In alkaline media, the rate-determining step (RDS) of HER is typically determined by the adsorption energy of reaction intermediates^49,50^. According to Sabatier’s principle, the optimal HER activity is achieved when the adsorption energy of the reaction intermediates is neither too high nor too low on the active sites^51,52^. Based on this perspective, we further investigated the influence of the coexistence of bridging S_2_^2−^ species and the Ru atoms in the amorphous layer on the adsorption energy of reactants. We first studied the undoped model with S_2_^2−^ group at the edge sites (the model was shown in Supplementary Fig. 34). The calculation results revealed that the hydrogen adsorption energies at the eight potential active sites were neither too strong (<−0.50 eV) nor too weak (>0.67 eV), which are all unfavorable for the HER^13,53^.

**Fig. 6:**
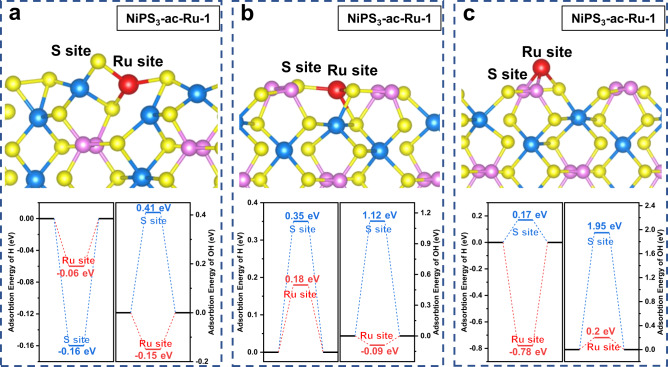
DFT calculation results of H^*^ and OH^*^ adsorption energy at Ru and adjacent S sites with typical configurations. **a** Ru atom is doped into the bridging S_2_^2−^ layer. **b** Ru atom is doped into other edge sites without bridging S_2_^2−^. **c** Ru atom is adsorbed onto the edge of NiPS_3_.

To explore the effects of Ru doping at the edge sites, three possible doping models with relatively stable structures were constructed and named NiPS_3_-ac-Ru-1, 2, and 3 (Supplementary Table 5). According to the calculation results, Ru doping led to the optimization of hydrogen adsorption energies to a favorable range (between −0.16 eV and 0.35 eV), which was superior to the undoped models (Fig. 6a, b). Nonetheless, if the Ru atom is simply adsorbed on the edges of NiPS_3_, the H* adsorption ability would remain too strong (about −0.78 eV), impeding the desorption of the H^*^ atom for the subsequent step (Fig. 6c). The adsorption ability of OH^*^ species is another crucial factor in evaluating the HER performance in alkaline electrolytes, as it affects the dissociation process of H_2_O molecules^5^. The ability of OH^*^ to adsorb onto the active sites of the catalyst can impact the overall reaction rate and efficiency. Therefore, on the basis of appropriate H^*^ adsorption energy, it is necessary to further consider the adsorption ability of active sites for OH^*^^54^. The DFT calculation results demonstrate that if the Ru atom was doped into the S_2_^2−^ layer (NiPS3-ac-Ru-1), the dissociated H* and OH* species would be adsorbed on S site (−0.16 eV) and adjacent Ru site (−0.15 eV) respectively. However, in the NiPS_3_-ac-Ru-2 and NiPS_3_-ac-Ru-3 models, both H^*^ and OH^*^ were found to preferentially adsorb onto the Ru sites, leading to competition for adsorption between the two species and potentially resulting in the poisoning of active sites^55^. Moreover, NiPS_3_-ac-Ru-1 demonstrated stronger adsorption ability for the Ru atom, resulting in a more stable structure (Supplementary Table 5). The DFT calculation results further proved that the formation of the S_2_^2−^ the group could effectively stabilize the doped Ru atom. Additionally, the separation of adsorption sites for H^*^ and OH^*^ can help prevent the poisoning of active sites while moderating the adsorption ability of reaction intermediates. These combined effects ultimately enhance the catalytic HER activity of Ru-NiPS_3_ NSs in alkaline electrolytes.

In summary, this study has demonstrated the significant role of the in situ formed amorphous layer and proposed an edge site decoration strategy to enhance the alkaline HER performance in layered catalysts. The in situ liquid electrochemical TEM was used to directly observe the amorphization in Ru-NiPS_3_, which is often overlooked in investigations of the origin of catalytic activity for metal phosphorus trichalcogenides. Theoretical calculations and experimental characterizations indicated that the formation of a surface amorphous layer with abundant bridging S_2_^2−^ species can stabilize the doped Ru atoms and reduce the leaching of functional groups during the HER process in alkaline electrolytes. Moreover, the doped Ru species located at the amorphous layer can modulate the adsorption energy of reaction intermediates and accelerate the water dissociation process, ultimately enhancing the alkaline HER activity. This work provides insights into the function of reconstructed amorphous layers in transition metal-based electrocatalysts and proposes a proper modulation strategy to improve the catalytic activity of layered materials.

## Methods

### Chemicals

All chemicals used in this study were of analytical grade and purchased from Aladdin Biochemical Technology Co., Ltd. in Shanghai, China. No further purification was carried out on any of the chemicals prior to use. Carbon cloth (CC) was obtained from Shanghai Hesen Electric Co., Ltd. and used in its as-received form.

### Synthesis of NiPS_3_ and Ru-NiPS_3_ electrodes

In this experiment, Ni precursors were grown on carbon cloth (CC) using a hydrothermal method^56^. Typically, 2 mmol Ni(NO_3_)_2_·6H_2_O, 10 mmol urea, and 5 mmol NH_4_F were dissolved in 40 mL of deionized (DI) water. After continuously stirring for 30 min, the solution was transferred into a 50 mL Teflon-lined stainless-steel autoclave. A piece of CC (with an area of 2 cm × 3 cm) was thoroughly cleaned with acetone, water, and ethanol to remove the possible contaminant on the surface. Then the CC was also transferred into the autoclave. The sealed autoclave was sealed and heated at 130 °C for 8 h, and then the CC was taken out. Afterward, the CC was cleaned with deionized water (DI) water and ethanol, and the precursor was dried in a vacuum at 60 °C for 8 h. To synthesize the NiPS_3_ NSs, 40 mg mixture of red phosphorus and sulfur powder, with a stoichiometric ratio of 1:3, was vacuum sealed in a quartz tube, together with the Ni precursor. Then the quartz tube was firstly heated at 300 °C for 30 min and then heated at 450 °C for 5 h, with a heating rate of 5 °C/min. After cooling to room temperature, the sample was washed with DI water and ethanol and dried in vacuum at 60 °C for further characterization. To synthesize the Ru-NiPS_3_ NSs, the Ni precursor was first immersed into the RuCl_3_ aqueous solution (3 mg ml^−1^) for different durations to finish the ion-exchange process. The resulting Ru-Ni precursor was then heated using a similar procedure to the synthesis of NiPS_3_ NSs. Since the doping amount of Ru is relatively low, the final loading of electrocatalysts on the electrode is about 1.5 mg cm^−2^. For comparison, NiPS_3_ single crystal was also fabricated with typical CVT methods following the description of previous work^57^.

### Materials characterizations

The crystalline phase and compositions were characterized by XRD (Rigaku SmartLab 9 kW) equipped with a λ = 1.54056 Å Cu Kα1 radiation source. Field emission scanning electron microscope (Thermo Fisher Scientific™ Helios 5 CX DualBeam™ Scanning Electron Microscope) was utilized to characterize the morphologies of the precursor and the final (Ru-)NiPS_3_ NSs. XPS spectra were collected on a Thermo Fisher Scientific Nexsa. The transmission electron microscope (JEOL, JEM-2100F; acceleration voltage, 200 kV), and double spherical aberration-corrected transmission electron microscope (Thermo Fisher Spectra 300, operated at 300 kV) were employed to investigate the morphologies, microstructures, and elements distribution. Dr. Probe was used for simulating STEM-HAADF images. Accelerating voltage, convergence semi-angle, and collection angle were set the same as the imaging, which were 300 kV, 15 mrad, and 35–200 mrad, respectively. Raman spectroscopy was conducted on a WITec, alpha300 R equipment (with a 532 nm laser). EPR spectra were obtained on a Bruker, A300-10-12 spectrometer. Atomic force microscopy (AFM) measurement was conducted on an AFM5300E system (HITACHI, Japan).

### In situ liquid electrochemical TEM characterization

To observe the in situ morphological evolution of Ru-NiPS_3_ NSs, a JEM-2100F electron microscope operated at 200 kV was used in conjunction with a liquid TEM holder (Protochips, Poseidon Select). The electron flux was calculated to be about 20 e^−^ Å^−2^ s^−1^. The liquid electrochemical chips (Protochips E-chips, ECT-45CR) are composed of two silicon chips, which are washed in acetone, methanol, and ethanol for 5 min, respectively, to remove the protective coating. Ru-NiPS_3_ ink was then dropped onto the silicon nitride (SiN_x_) window, and the chips were further cleaned with Ar/O_2_ plasma for 30 s. The in situ observation was conducted in an alkaline media solution (0.1 M NaOH), and a Gamry 600+ potentiostat (Gamry, Warminster, PA) was used to provide a constant current of -5 nA vs. Pt during the whole observation. The electrolyte flow rate is controlled at 200 μL h^−1^ to avoid damaging the SiN_x_ window. In order to obtain clear TEM images with good spatial resolution, the samples in these in situ liquid TEM experiments were imaged within a thin liquid layer. It should be noted that the applied experimental conditions in these studies may not perfectly replicate those of a realistic electrochemical cell. As a result, there may be minor differences between the results obtained from in situ and ex situ measurements.

### Electrochemical measurements

All electrochemical measurements were conducted using a typical three-electrode cell with a CHI 760E electrochemical workstation (CH Instruments, Inc. Shanghai). The as-systemized electrode, Hg/HgO electrode, and graphite rod (Alfa Aesar, 99.9995%) were used as the working electrode, reference electrode, and counter electrode, respectively. Before the LSV test in 1 M KOH electrolyte, all electrodes were activated by the cyclic voltammetry (CV) technique for 100 cycles to obtain stable LSV curves. The scan rate is 50 mV s^−1^, within the potential range from −0.8 V vs. Hg/HgO to −1.5 V vs. Hg/HgO, and the total activation time is about 1 h. To avoid the influence of the Ru species dissolved in the electrolyte during the activation process, the electrode was replaced immediately when the activation process is finished. LSV curves were then obtained with a scan rate of 2 mV s^−1^. In this work, all potentials were converted to RHE with the equation E_RHE_ = E_Hg/HgO_ + 0.098 V + 0.059 × pH. EIS measurements were carried out within a frequency range of 10^6 ^Hz to 10^−2 ^Hz, and the charge transfer resistance (*R*_ct_) obtained by fitting the EIS data was used for the *iR* correction.

### Calculation details

To investigate the geometries and electronic properties of Ru-doped NiPS_3_ materials, spin-polarized density functional theory (DFT) calculations were conducted using the Vienna ab initio Simulation Package (VASP) program package^58,59^ with the projector augmented wave (PAW)^60^. The exchange-correlation interactions were described using the Perdew, Burke, and Ernzernhof (PBE) functional^60^ with the generalized gradient approximation (GGA)^61^. The kinetic energy cutoff for the plane-wave basis set was set to 400 eV, and the distance of the vacuum layer was greater than 20 Å to prevent interlayer interactions. To correct for van der Waals interactions on the surface, the DFT-D3 scheme of Grimme was applied^62^. The electronic SCF tolerance was set to 10^−4^ eV. Fully relaxed geometries and lattice constants were obtained by optimizing all atomic positions until the Hellmann–Feynman forces were less than 0.04 eV/Å. The structural optimizations used a gamma-centered Monkhorst–Pack scheme with k-point samplings of 2 × 1 × 1^61^. To convert the calculated DFT adsorption energies (Δ*E*) into Gibbs free energies (Δ*G*) for H (0.24 eV)^63^ and OH* (0.29 eV)^64^, entropic (TS) and zero-point energy (ZPE) corrections were applied to the adsorbed species.

### Supplementary information

Supplementary Information

Peer Review File

Description of Additional Supplementary Files

Supplementary Movie 1

Supplementary Movie 2

Supplementary Movie 3

## Data Availability

The data that support the findings of this study are available from the corresponding author upon reasonable request.
