# Supplementary material for: Unraveling and leveraging in situ surface amorphization for enhanced hydrogen evolution reaction in alkaline media
Source: Nat Commun. 2023 Oct 13;14:6462. doi: 10.1038/s41467-023-42221-6 (PMC10575887; doi:10.1038/s41467-023-42221-6)
Supplement: Supplementary file 1 — Supplementary Information [file 41467_2023_42221_MOESM1_ESM.pdf]

Supplementary Information

Unraveling and Leveraging *in situ* Surface Amorphization for Enhanced Hydrogen Evolution Reaction in Alkaline Media

Qiang Fu,[1, †] Lok Wing Wong,[1, †] Fangyuan Zheng,[1] Xiaodong Zheng,[1] Chi Shing Tsang,[1] Ka Hei Lai,[1] Wenqian Shen,[1] Thuc Hue Ly,[2, 3, 4 *] Qingming Deng,[5 *] & Jiong Zhao[1, 6 *]

[1] *Department of Applied Physics, The Hong Kong Polytechnic University, Kowloon, China.*

[2] *Department of Chemistry and Center of Super-Diamond & Advanced Films (COSDAF), City University of Hong Kong, Kowloon, China.*

[3] *Department of Chemistry and State Key Laboratory of Marine Pollution, City University of Hong Kong, Hong Kong, China*

[4] *City University of Hong Kong Shenzhen Research Institute, Shenzhen, China.*

[5] *Phyics Department and Jiangsu Key Laboratory for Chemistry of Low-Demensional Materials, Huaiyin Normal University, Huaian, China*

[6] *The Hong Kong Polytechnic University Shenzhen Research Institute, Shenzhen, China.*

**emails:* thuchly@cityu.edu.hk(L.T.H.); qingmingdeng@gmail.com(D.Q.); jiongzhao@polyu.edu.hk(Z.J.)

† These authors contribute equally: Qiang Fu and Lok Wing Wong

Table of Contents

Supplementary Fig. 1………………………………………………………………..………..S4

Supplementary Fig. 2……………………………………………..……………………..S5

Supplementary Fig. 3……………………………………………………..…………..S6

Supplementary Fig. 4……………………………………………………..…………..S7

Supplementary Fig. 5………………………………………………………..………..S8

Supplementary Fig. 6…………………………………………………………..……..S9

Supplementary Fig. 7…………………………………………………..…………....S10

Supplementary Fig. 8…………………………………………………..…………....S11

Supplementary Fig. 9…………………………………………………………..…..…..S12

Supplementary Fig. 10………………………………………………………………..…..S13

Supplementary Fig. 11…………………………………………………………………....S14

Supplementary Fig. 12…………………………………………………………...………S15

Supplementary Fig. 13……………………………………………………….………...S16

Supplementary Fig. 14………………………………………………………….………..S17

Supplementary Fig. 15…………………………………………………………...………S18

Supplementary Fig. 16……………………………………………………………..…...S19

Supplementary Fig. 17…………………………………………………………..……..S20

Supplementary Fig. 18…………………………………………………………..……..S21

Supplementary Fig. 19…………………………………………………………..……..S22

Supplementary Fig. 20…………………………………………………………..…..S23

Supplementary Fig. 21………………………………………………………………....S24

Supplementary Fig. 22……………………………………………………………..……..S25

Supplementary Fig. 23……………………………………………………………..…..S26

Supplementary Fig. 24…………………………………………………………………...S27

Supplementary Fig. 25……………………………………………………………..…..S28

Supplementary Fig. 26……………………………………………………………..…..S29

Supplementary Fig. 27……………………………………………………………..…..S30

Supplementary Fig. 28……………………………………………………………..…..S31

Supplementary Fig. 29……………………………………………………………..…..S32

Supplementary Fig. 30……………………………………………………………..…..S33

Supplementary Fig. 31……………………………………………………………..…..S34

Supplementary Fig. 32……………………………………………………………..…..S35

Supplementary Fig. 33……………………………………………………………..…..S36

Supplementary Fig. 34……………………………………………………………..…..S37

Supplementary Table 1……………………………………………………………...…….S38

Supplementary Table 2…………………………………………………………......…. S39

Supplementary Table 3……………………………………………………………...…….S40

Supplementary Table 4……………………………………………………………...…….S41

Supplementary Table 5……………………………………………………………...…….S42

References…………………………………………………………………………...…S43

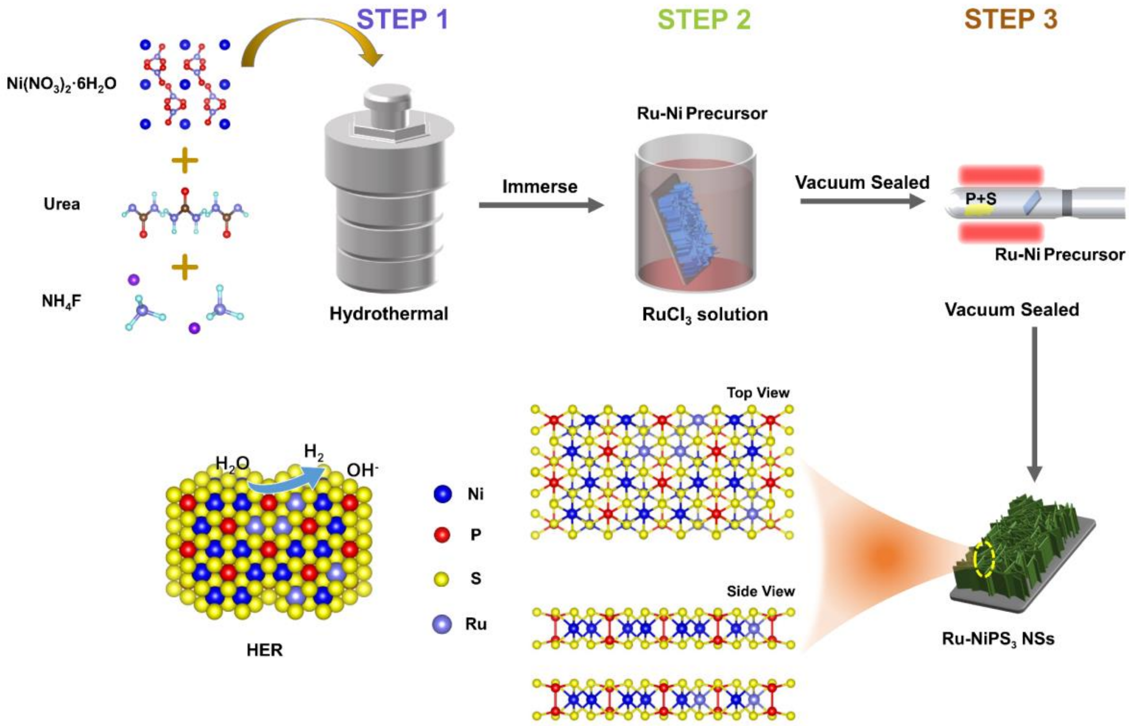

Supplementary Fig. 1. Schematic illustration of synthetic procedures for Ru-$NiPS_3$ NSs.

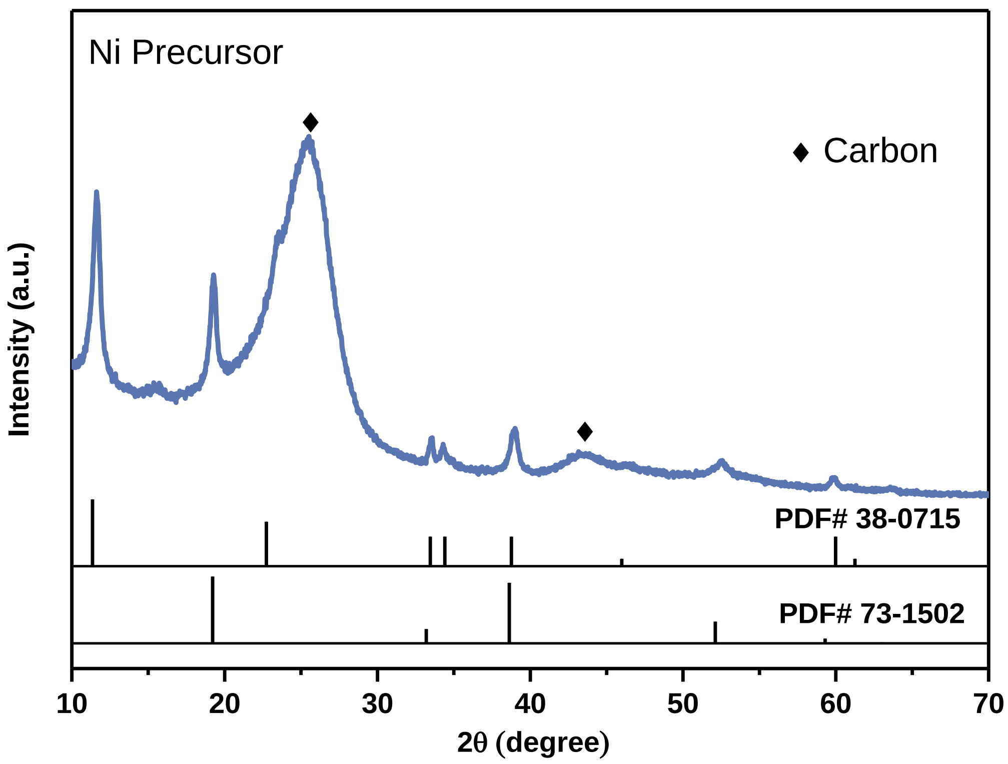

Supplementary Fig. 2. XRD pattern of the Ni precursor prepared with hydrothermal method

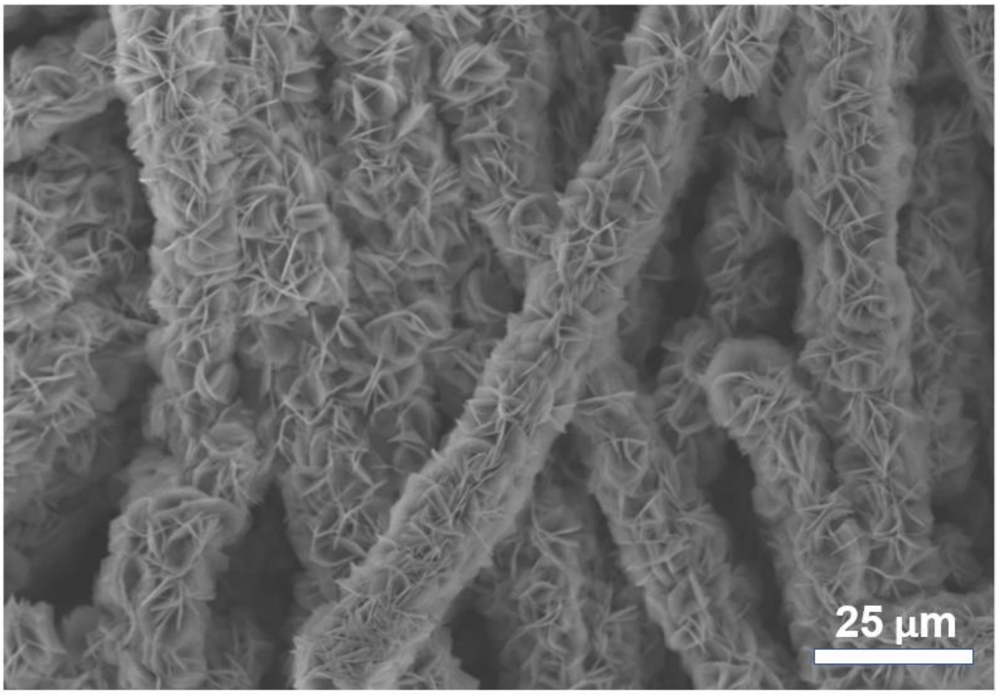

Supplementary Fig. 3. SEM image of the Ni precursor

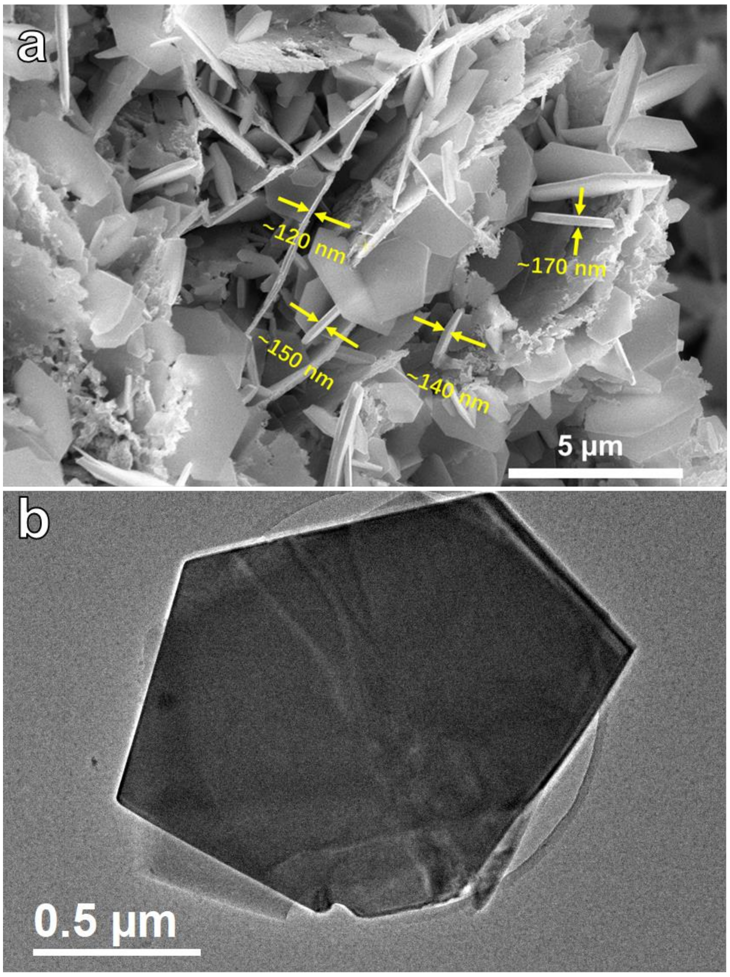

Supplementary Fig. 4. (a) SEM and (b) TEM image of Ru-$NiPS_3$ NSs (dipping for 16h)

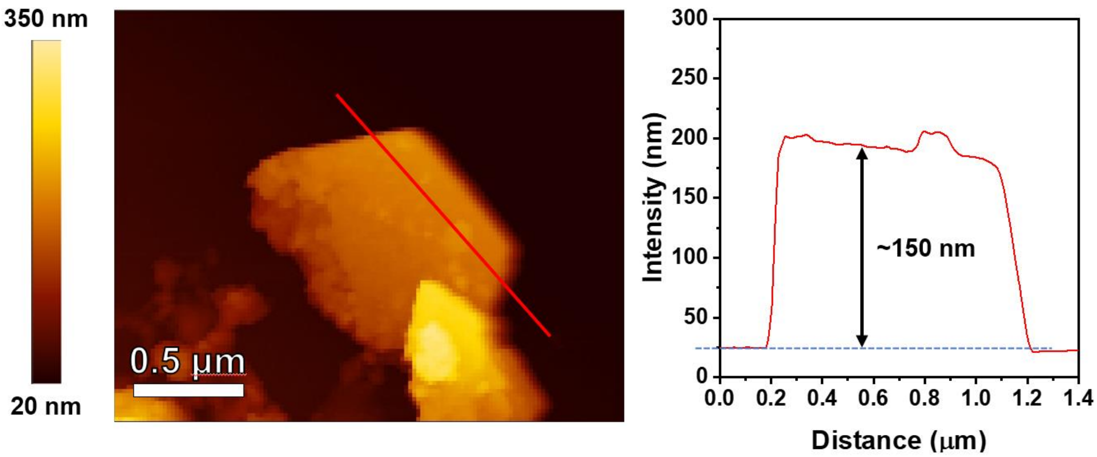

Supplementary Fig. 5 AFM image and corresponding height profiles. The results demonstrated that the typical thickness of the nanosheets is around 150 nanometers.

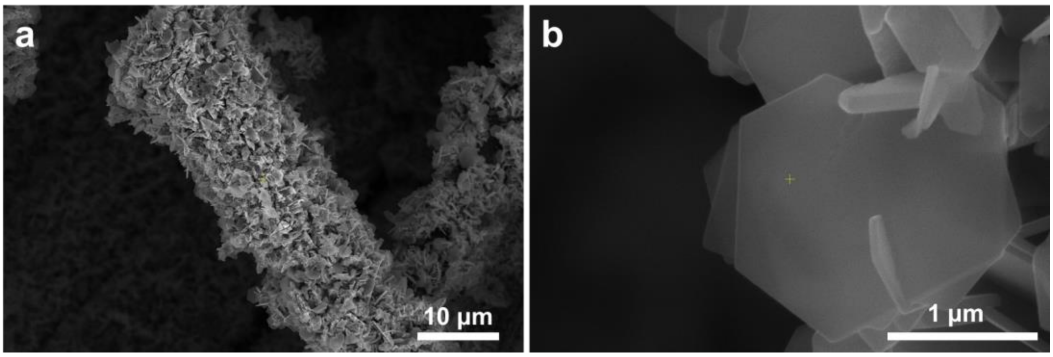

Supplementary Fig. 6. SEM image of the $NiPS_3$ sample prepared without dipping in Ru solution

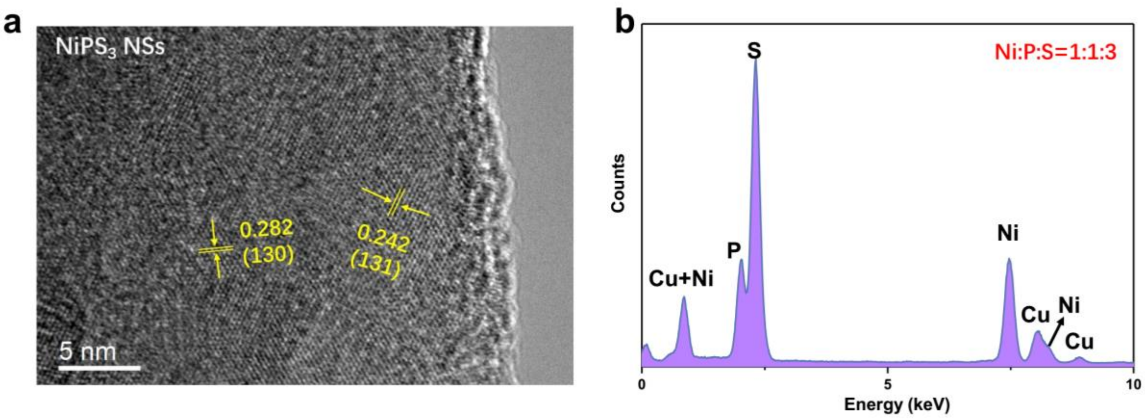

Supplementary Fig. 7. (a) HRTEM image and (b) the corresponding EDS spectrum for NiPS$_3$ NSs

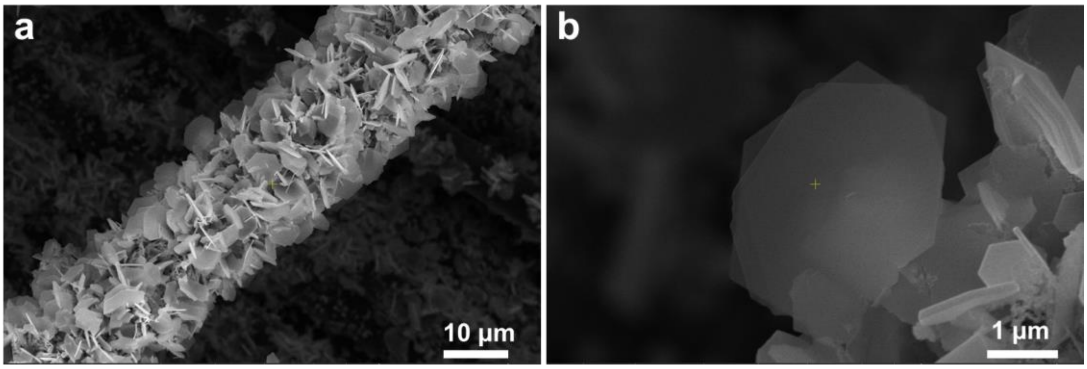

Supplementary Fig. 8. SEM image of the $NiPS_3$ sample prepared dipping in Ru solution for 0.5 h

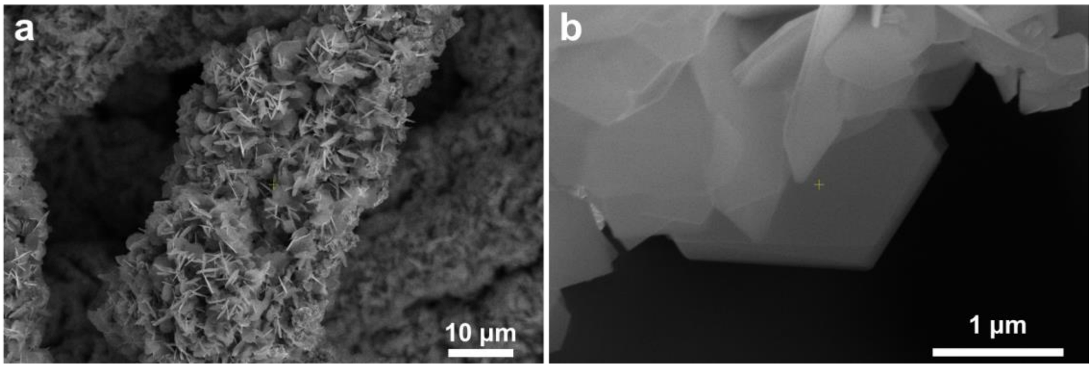

Supplementary Fig. 9. SEM image of the $NiPS_3$ sample dipping in Ru solution for 2 h

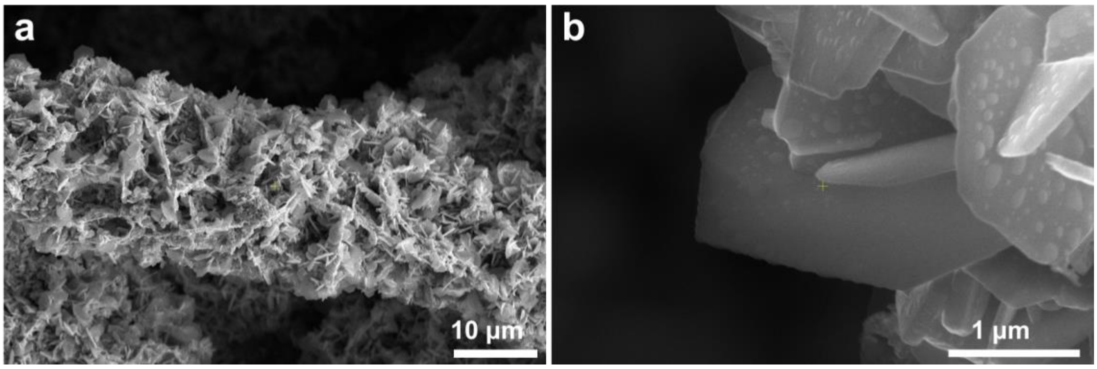

Supplementary Fig. 10. SEM image of the $NiPS_3$ sample dipping in Ru solution for 4 h

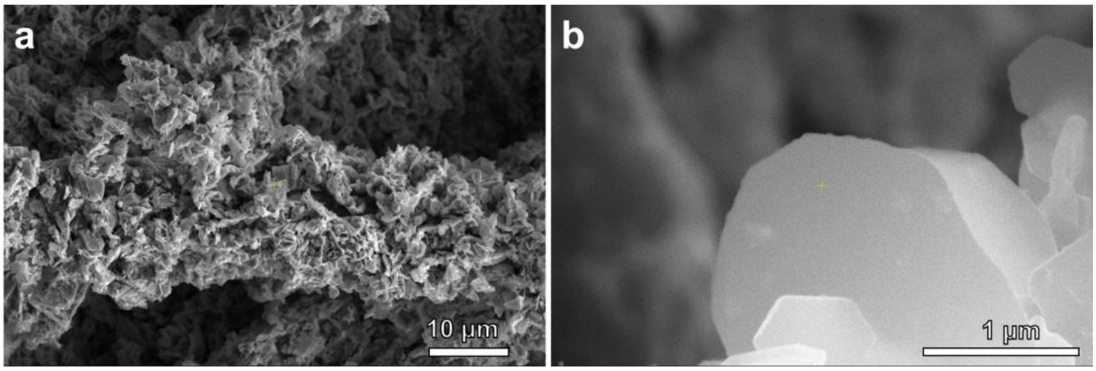

Supplementary Fig. 11. SEM image of the $NiPS_3$ sample dipping in Ru solution for 20 h

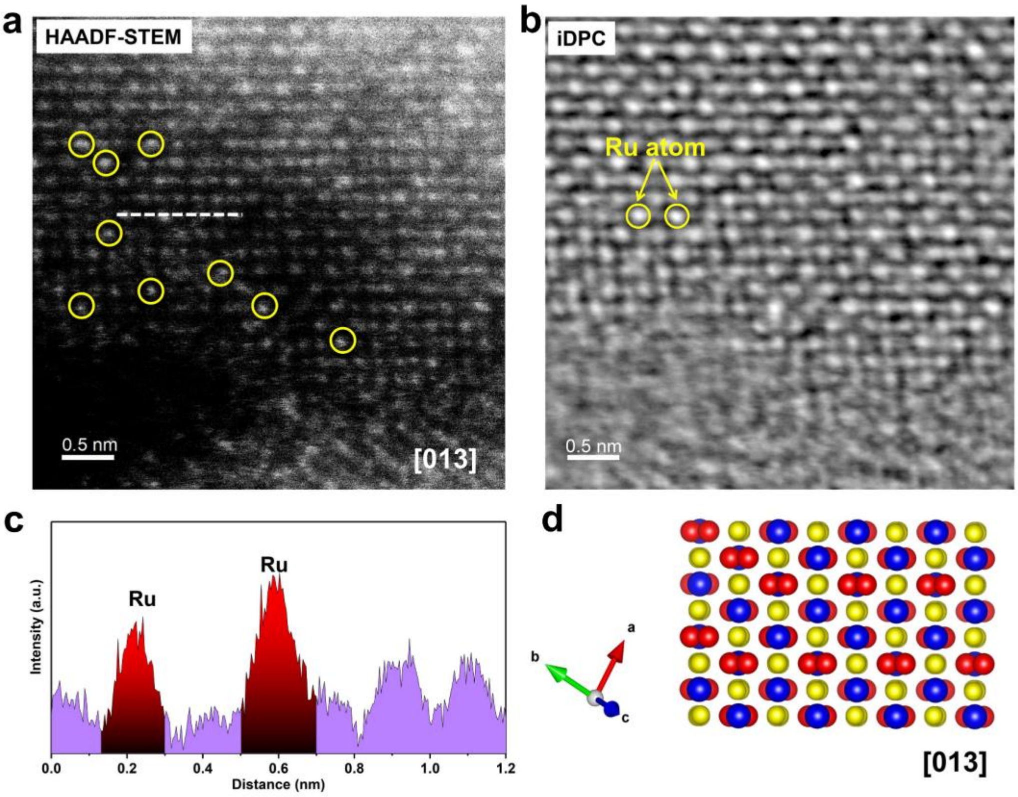

Supplementary Fig. 12. (a) AC-HAADF stem image and (b) the corresponding iDPC image of Ru-$NiPS_3$ along [013] zone axis (Ru atoms are highlighted by yellow circles). (c) the corresponding intensity line profiles taken along the white line in (a), which demonstrated that the Ru atom replaced the Ni atom in the $NiPS_3$ NSs. (d) showed the Crystal structure of $NiPS_3$ along the [013] zone axis.

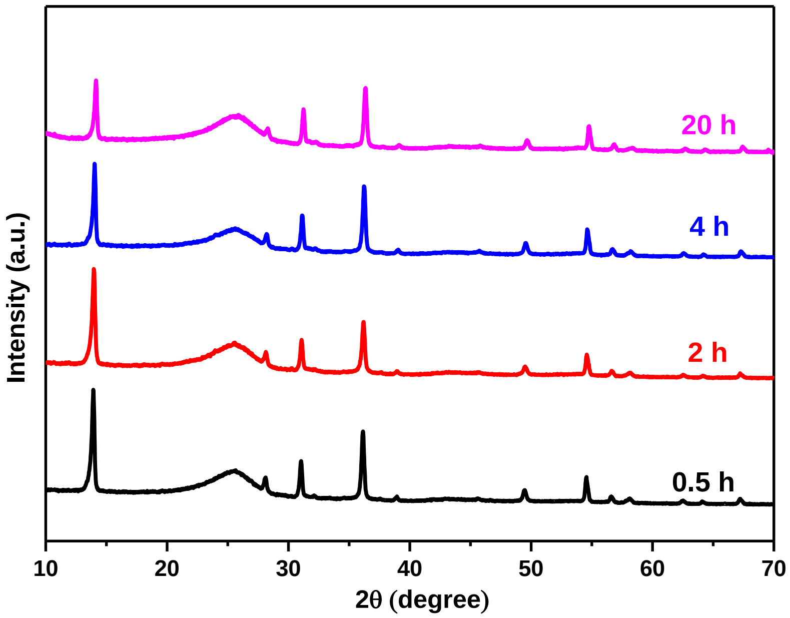

Supplementary Fig. 13. XRD patterns of various Ru-$NiPS_3$ NSs with different dipping times in $RuCl_3$ solution.

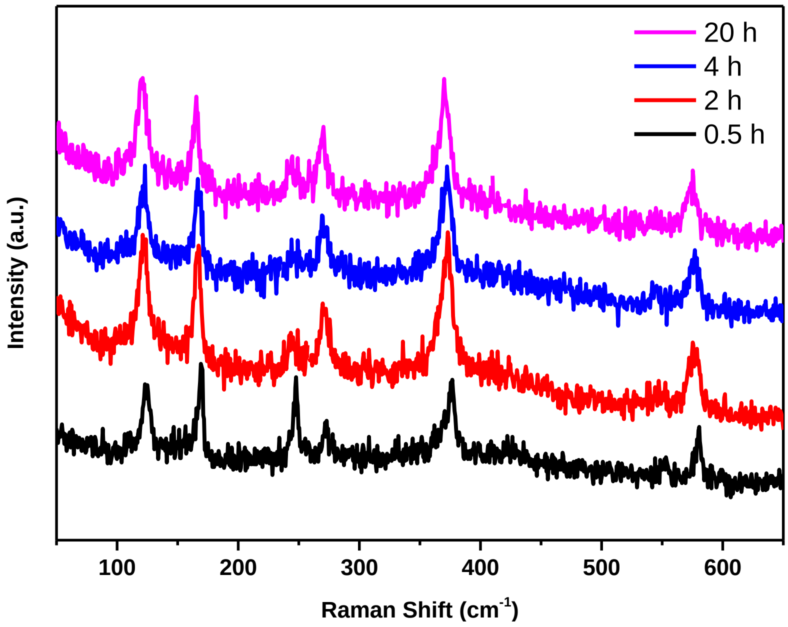

Supplementary Fig. 14. Raman spectra of various Ru-$NiPS_3$ NSs with different dipping times in $RuCl_3$ solution

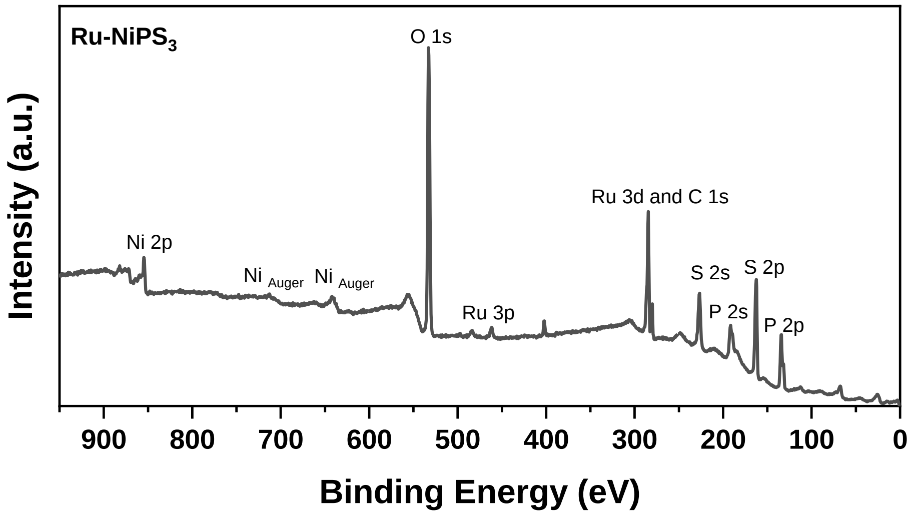

Supplementary Fig. 15. XPS survey spectrum for Ru-NiPS$_3$ NSs

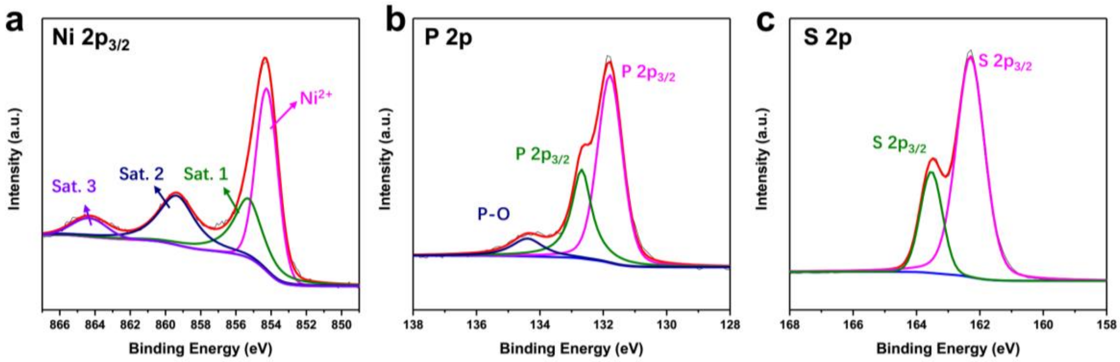

Supplementary Fig. 16. XPS spectra of $NiPS_3$ NSs. (a) Ni $2p_{3/2}$, (b) P 2p, and (c) S 2p

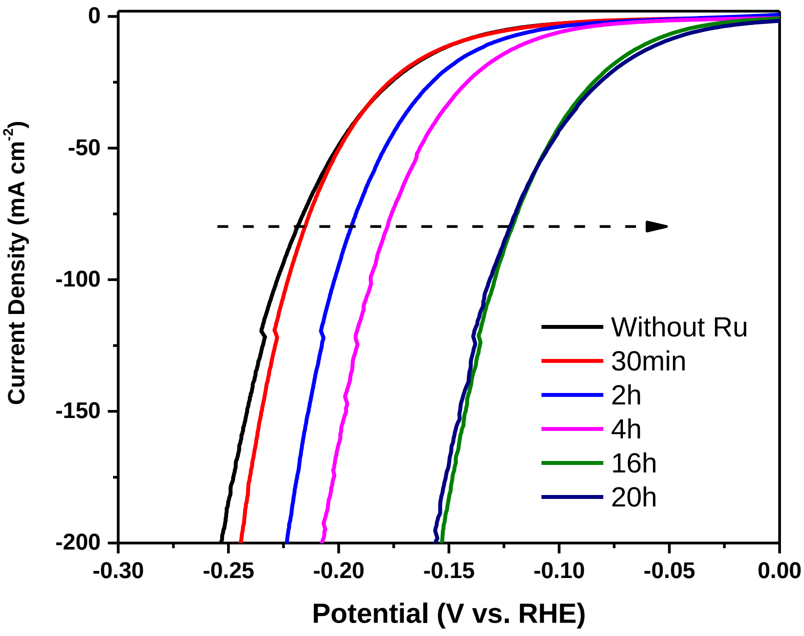

Supplementary Fig. 17. Comparison of HER performance for different samples in 1M KOH (pH = 14). The mass loading of the electrocatalysts is ~1.5 mg cm^{-2}, and the solution resistance is ~3.0 Ω.

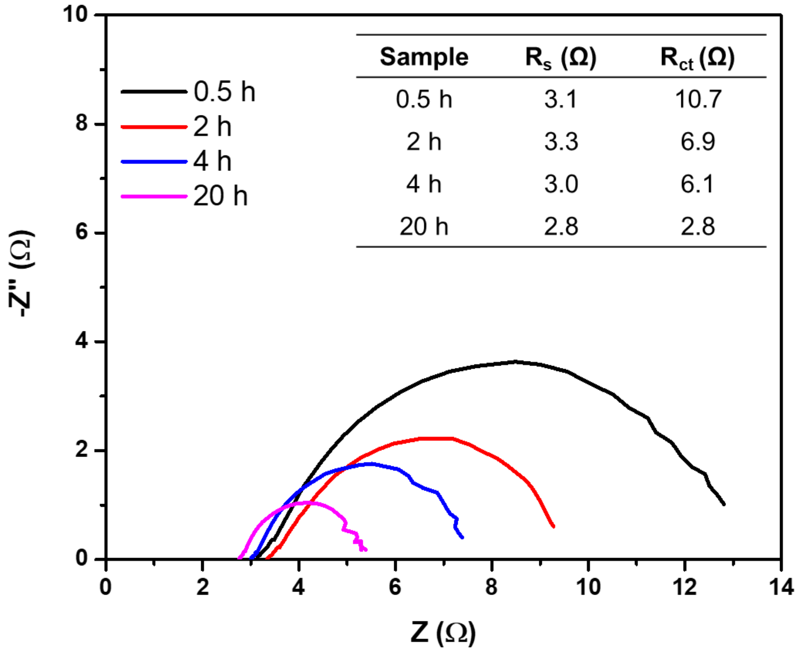

Supplementary Fig. 18. Nyquist plots for samples dipping in $RuCl_3$ solution with different times

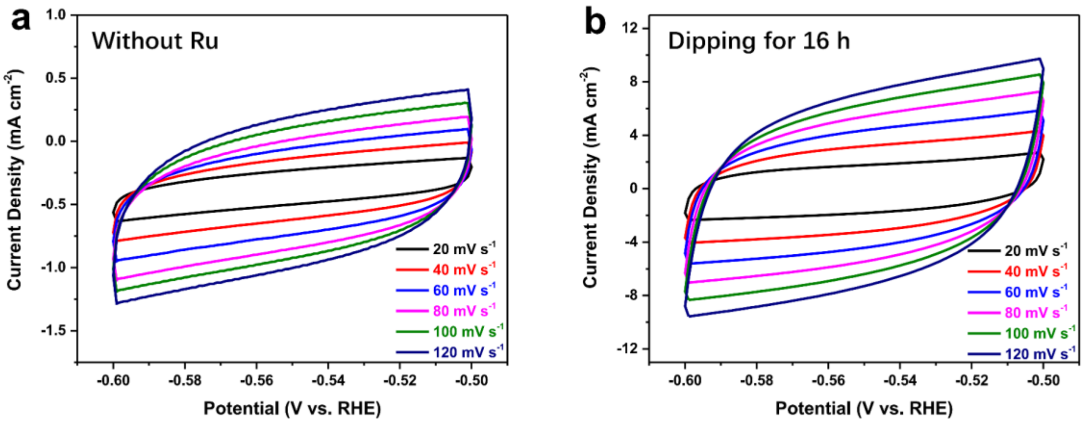

Supplementary Fig. 19. CV curves for C_{dl} of the samples (a) without Ru and (b) dipping for 16 h at the scan rates from 20 mV/s to 120 mV/s

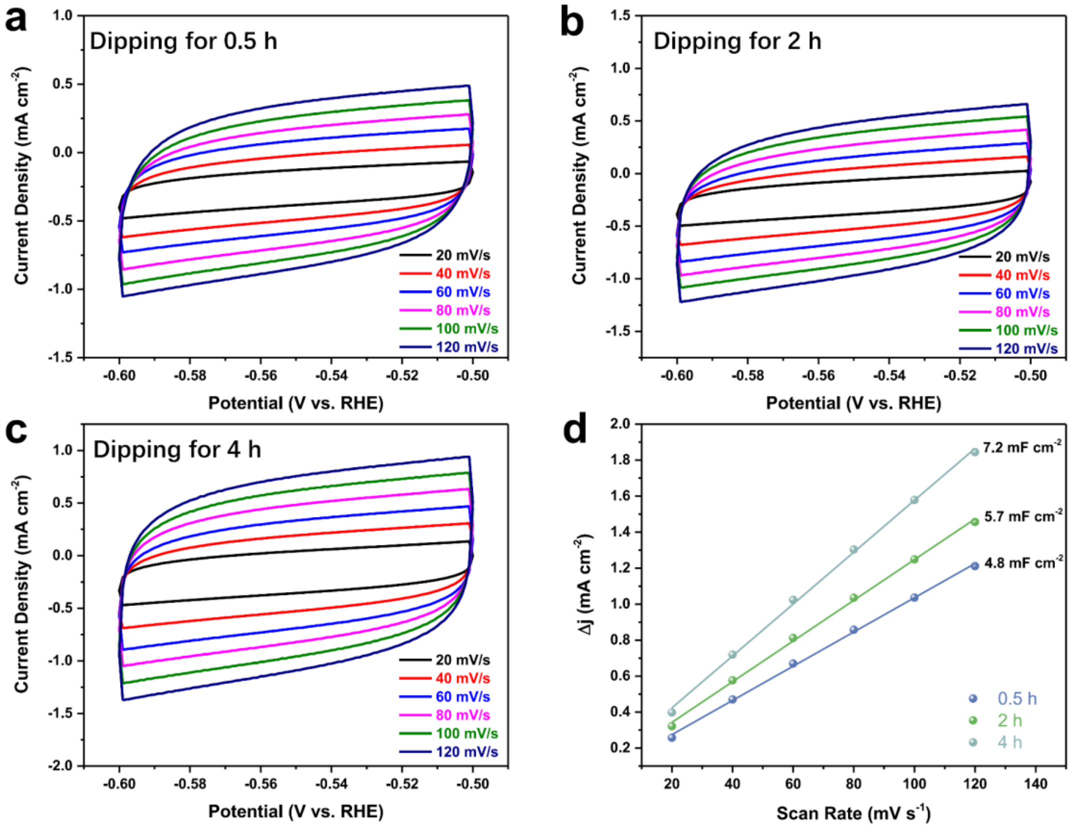

Supplementary Fig. 20. CV curves for the samples dipping in $RuCl_3$ solution for (a) 0.5 h, (b) 2 h, (c) 4 h, and the corresponding C_{dl} values

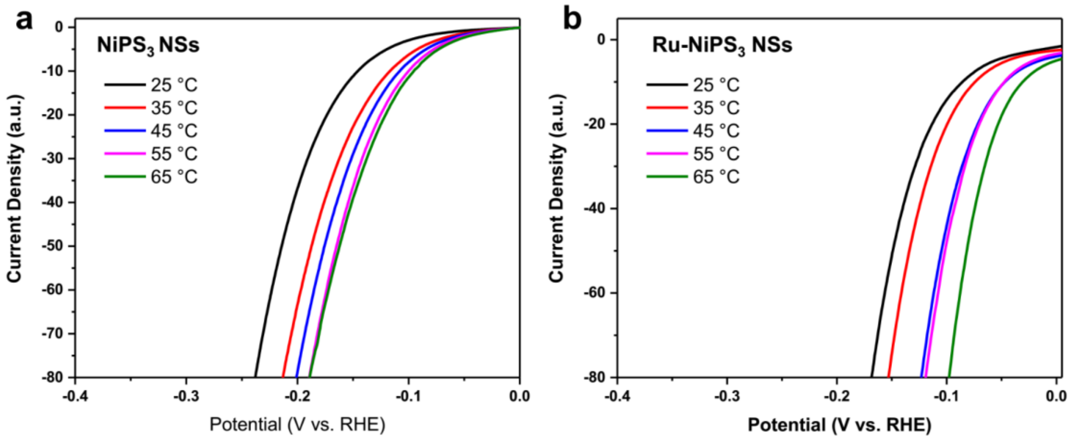

Supplementary Fig. 21. LSV curves of (a) $NiPS_3$ and (b) Ru-$NiPS_3$ were obtained under different temperatures in 1 M KOH solution (Ph = 14). The mass loading of the electrocatalysts is ~1.5 mg cm^{-2}, and the solution resistance is ~3.0 Ω. These results were used to calculate the apparent activation energy (E_{app}) and the pre-exponential factor (A_{app}) for both catalysts, as shown in Supplementary Fig. 21.

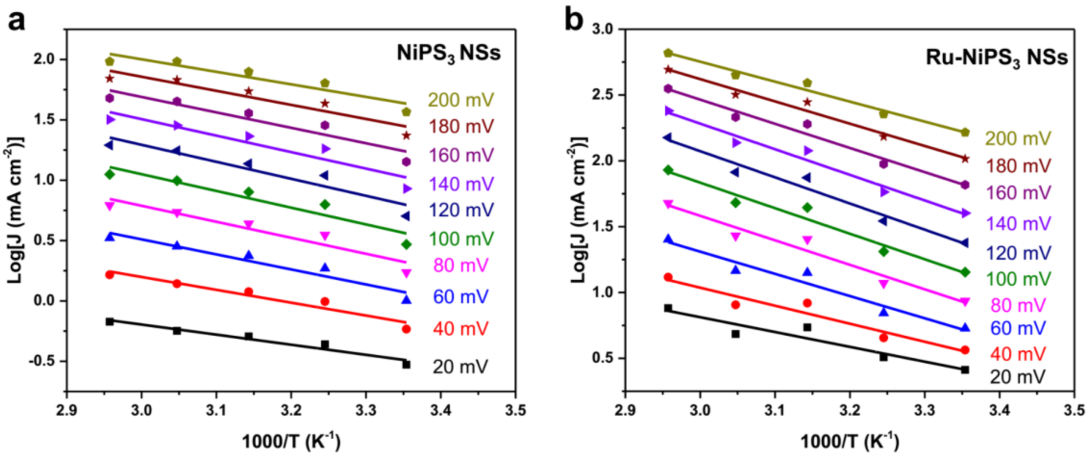

Supplementary Fig. 22. The logarithm of the catalytic current density plotted against 1000 times the reciprocal of the temperature (in Kelvin) to extract the apparent activation energy (E_{app}) and the pre-exponential factor (A_{app}) of the HER on (a) $NiPS_3$ and (b) Ru-$NiPS_3$ at fixed overpotentials using Arrhenius plots[3].

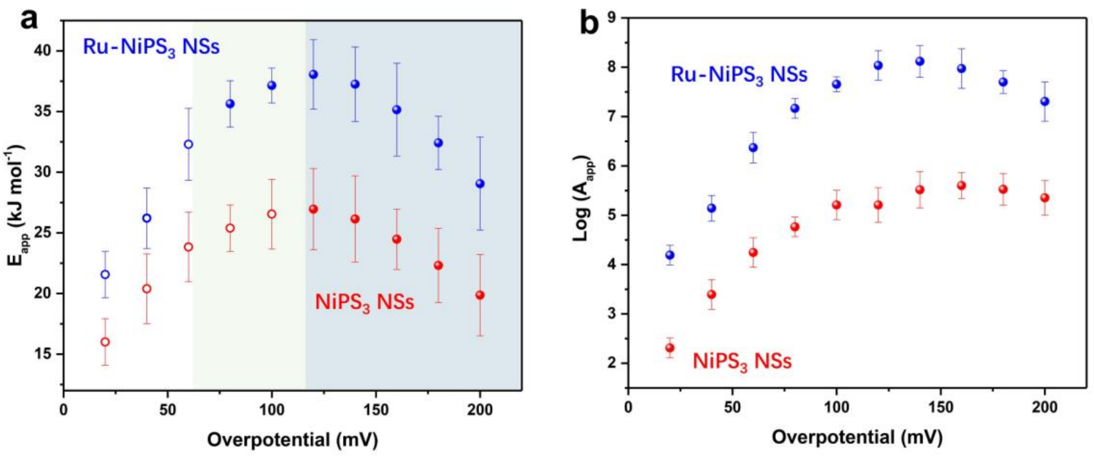

Supplementary Fig. 23. (a) E_{app} and (b) The logarithm of pre-exponential factor A_{app} derived from the intercept during the extraction of E_{app} for $NiPS_3$ and Ru-$NiPS_3$ at fixed overpotentials using the Arrhenius plots, as shown in Supplementary Fig. 20.

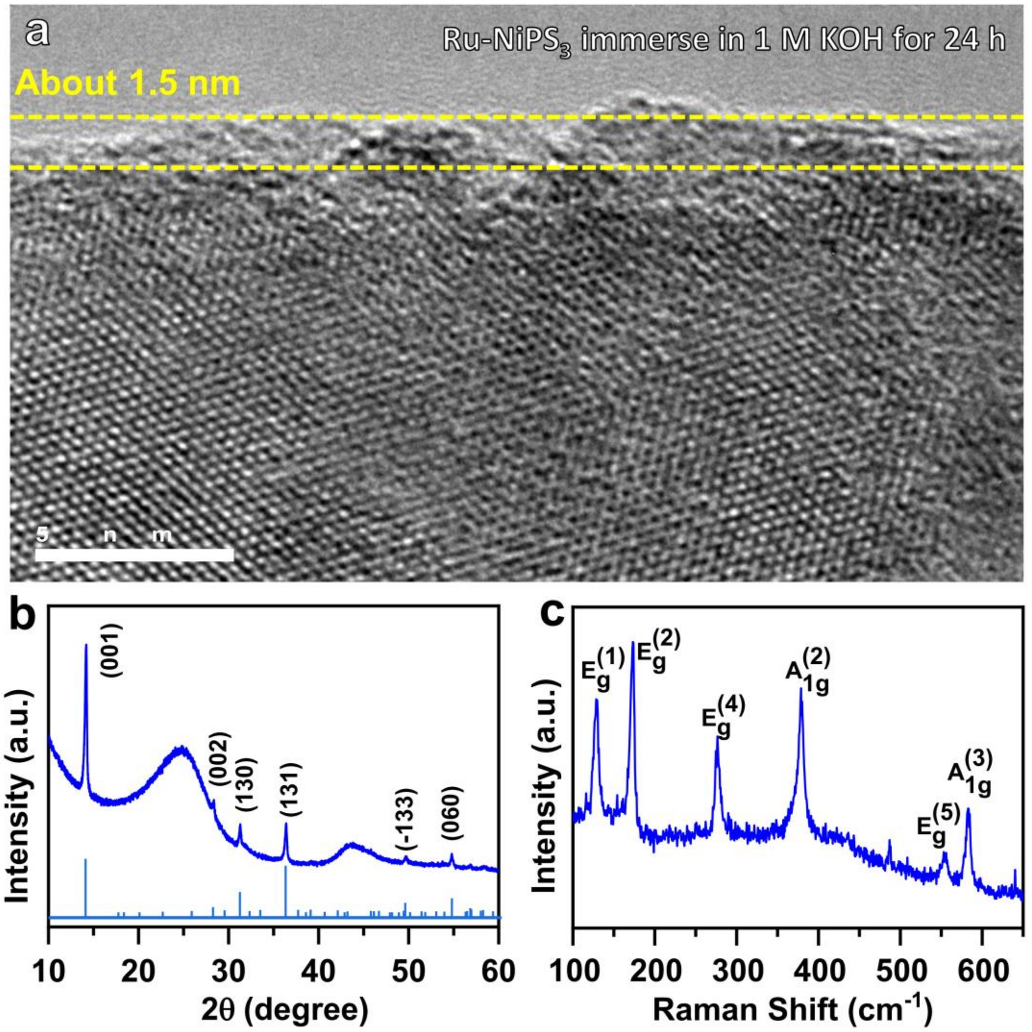

Supplementary Fig. 24. (a) HRTEM image, (b) XRD pattern, and (c) Raman spectrum of Ru-$NiPS_3$ NSs after immersing in 1 M KOH solution for 24h.

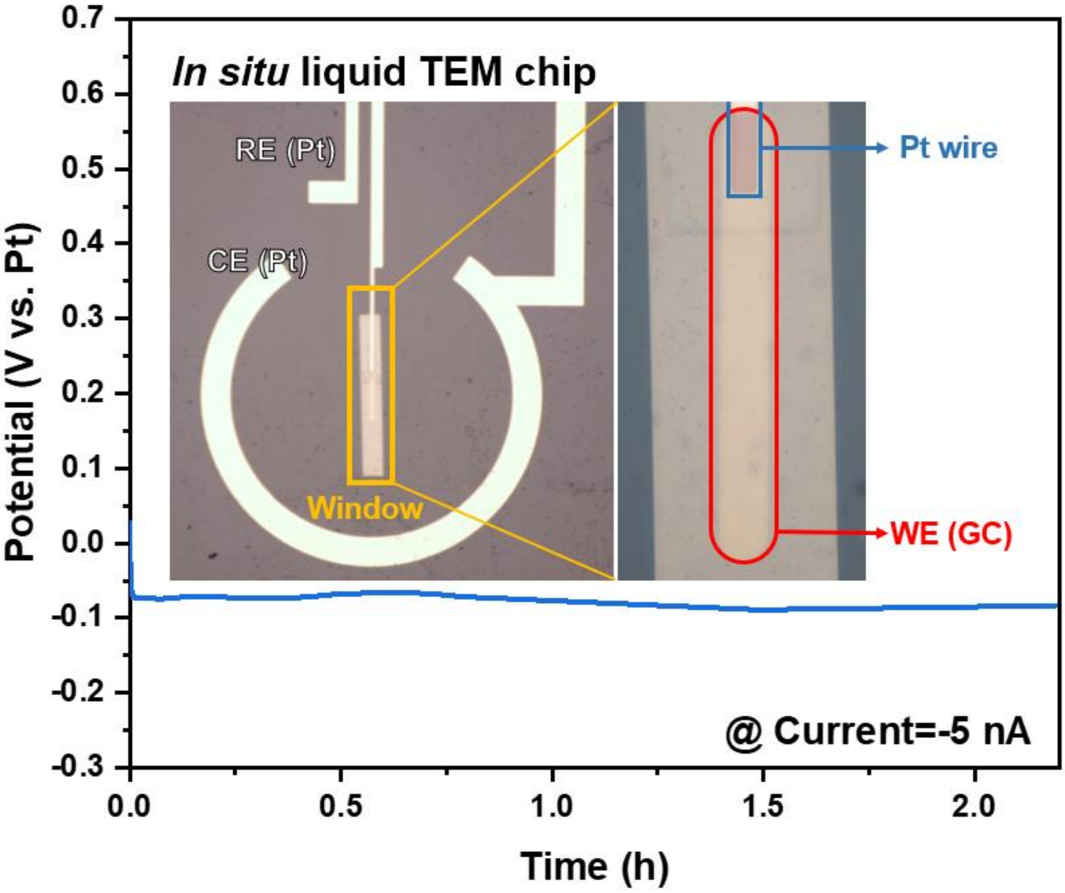

Supplementary Fig. 25. The chronopotentiometry data obtained during *in situ* liquid TEM measurements. The accompanying inset illustrates the structure of the liquid TEM chip.

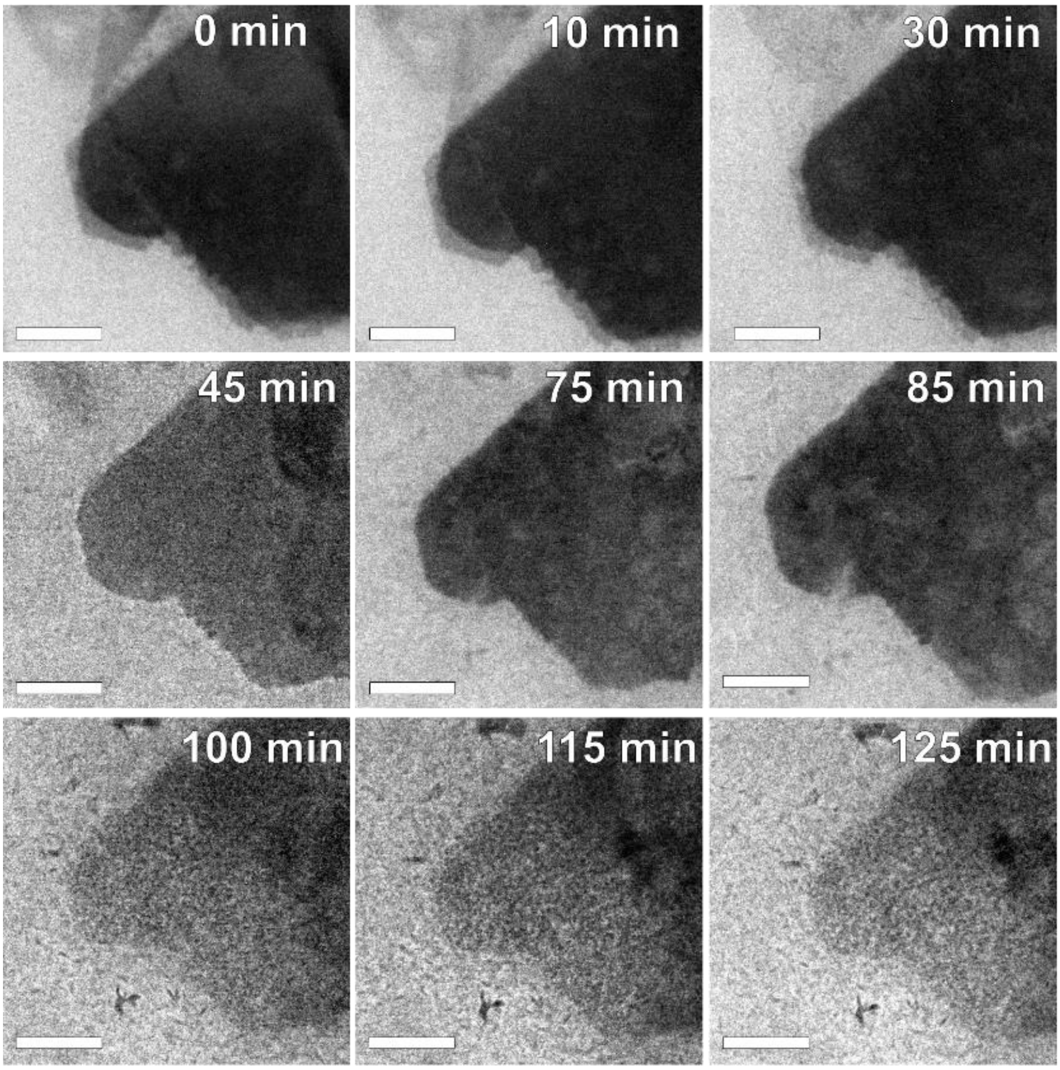

Supplementary Fig. 26. *In situ* TEM sequential images showing the evolution of the Ru-$NiPS_3$ NSs on GC electrode in about 2h (scale bar: 0.2 μm).

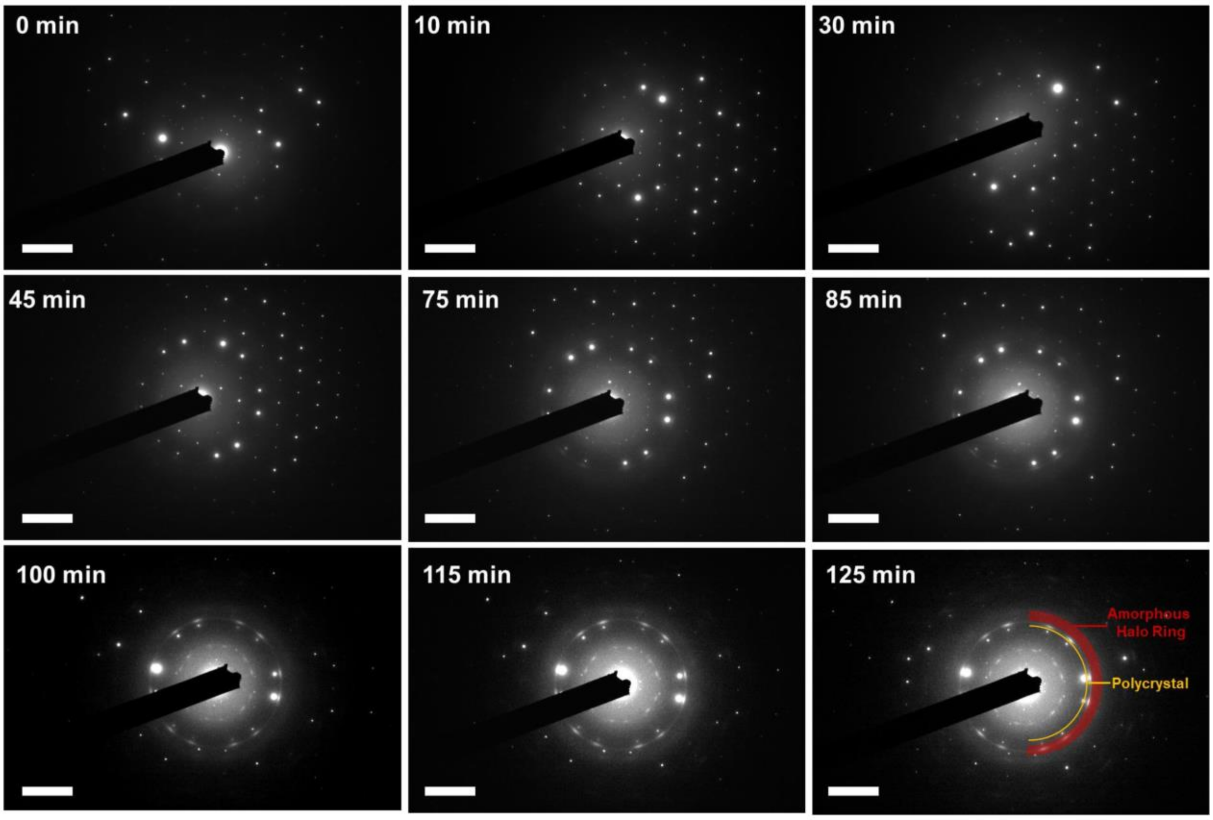

Supplementary Fig. 27. *In situ* sequential SAED pattern showing the evolution of the Ru-$NiPS_3$ NSs on GC electrode in 125 min (scale bar: 5 1/nm).

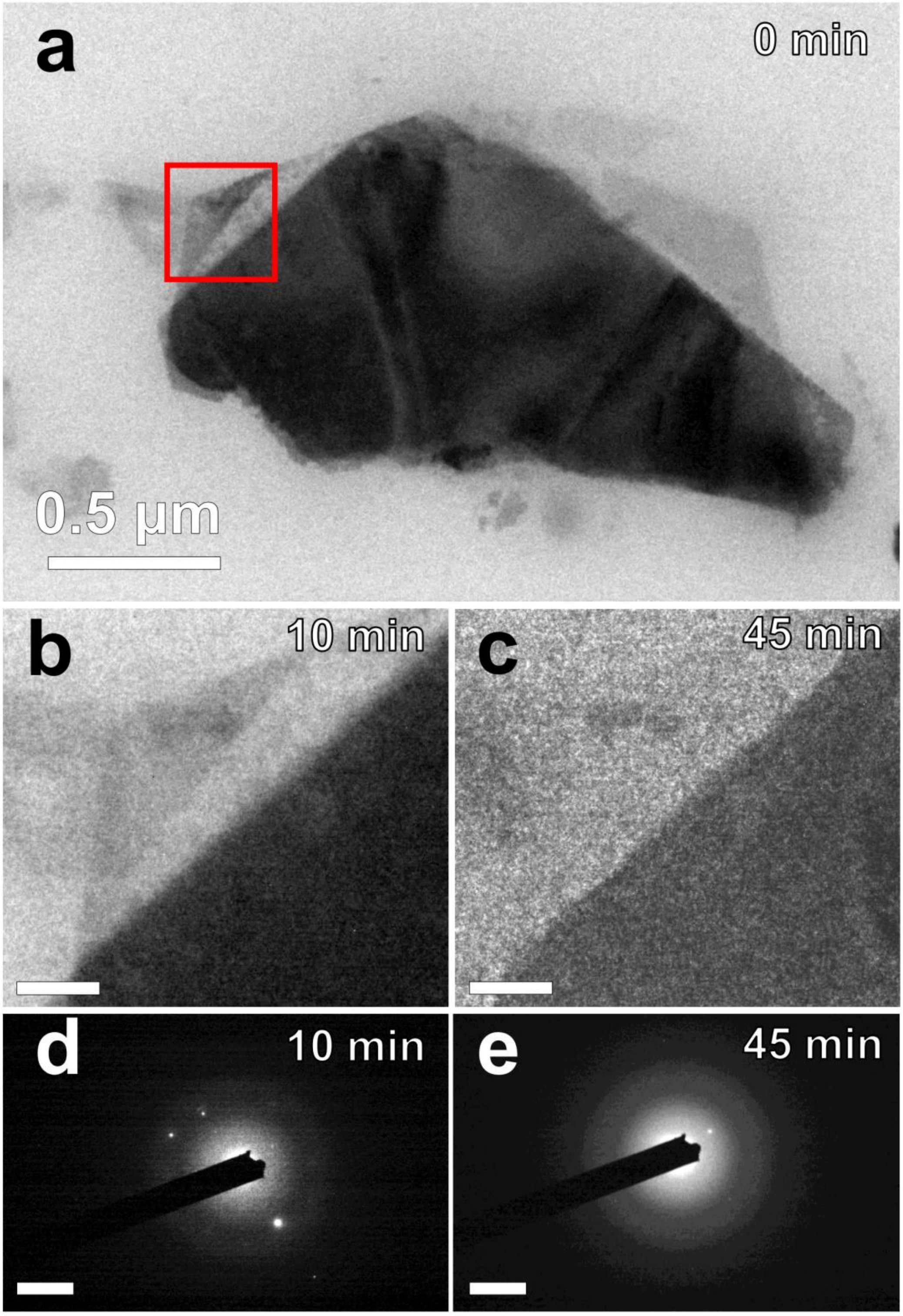

Supplementary Fig. 28. (a) TEM image of the initial Ru-$NiPS_3$ NSs without electrochemical reaction. (b) and (c) are the TEM image of the select area in (a) (red square) after 10 min and 45 min reaction, respectively (scale bar: 0.1 µm). (d) and (f) are the corresponding SAED patterns for (b) and (c), respectively (scale bar: 5 1/nm).

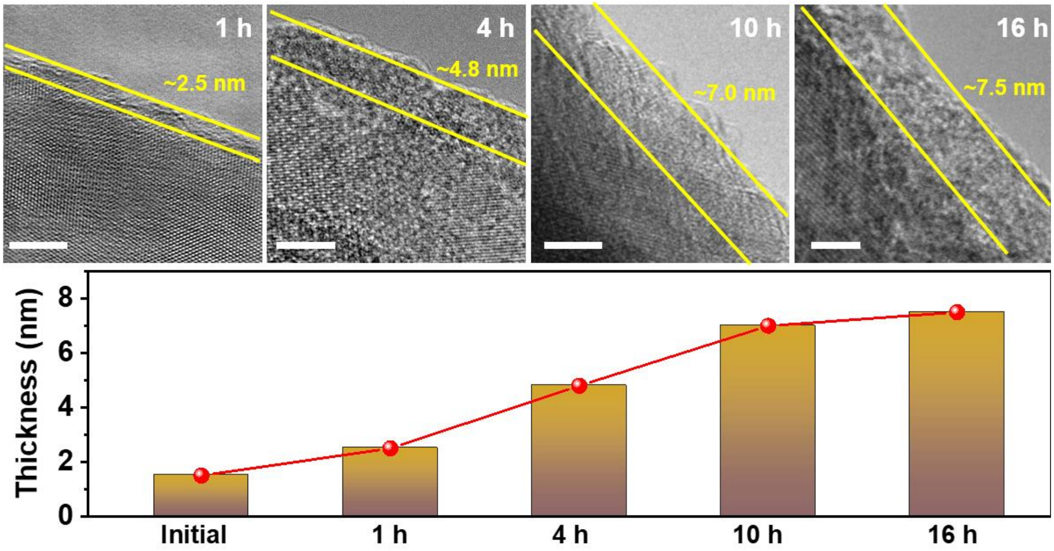

Supplementary Fig. 29. *Ex situ* TEM images of the representative sample Ru-$NiPS_3$ NSs and the estimated thickness of amorphous layer after stability tests for varying durations (1 h, 4 h, 10 h, and 16h reaction duration; scale bar: 5 nm). The electrochemical test was operated in 1 KOH solution, with a constant current density of -100 mA cm^{-2}. The results demonstrated that as the reaction progresses, the thickness of the amorphous layer around the nano flakes tends to stabilize (~8 nm), thereby stabilizing the overall structure of the catalyst.

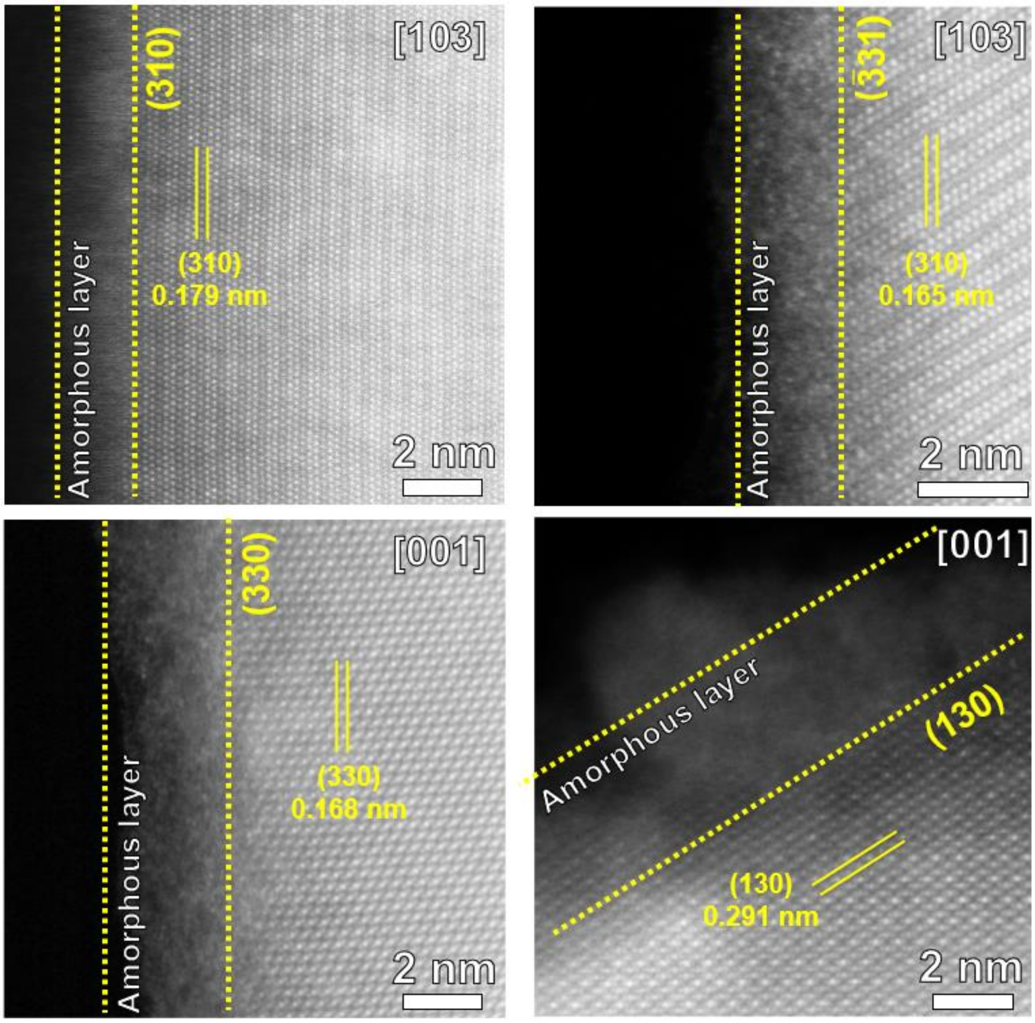

Supplementary Fig. 30. HDDF-STEM images of Ru-$NiPS_3$ alone [001] and [103] zone axis, which showed that the amorphization process is independent of the crystal plane orientation.

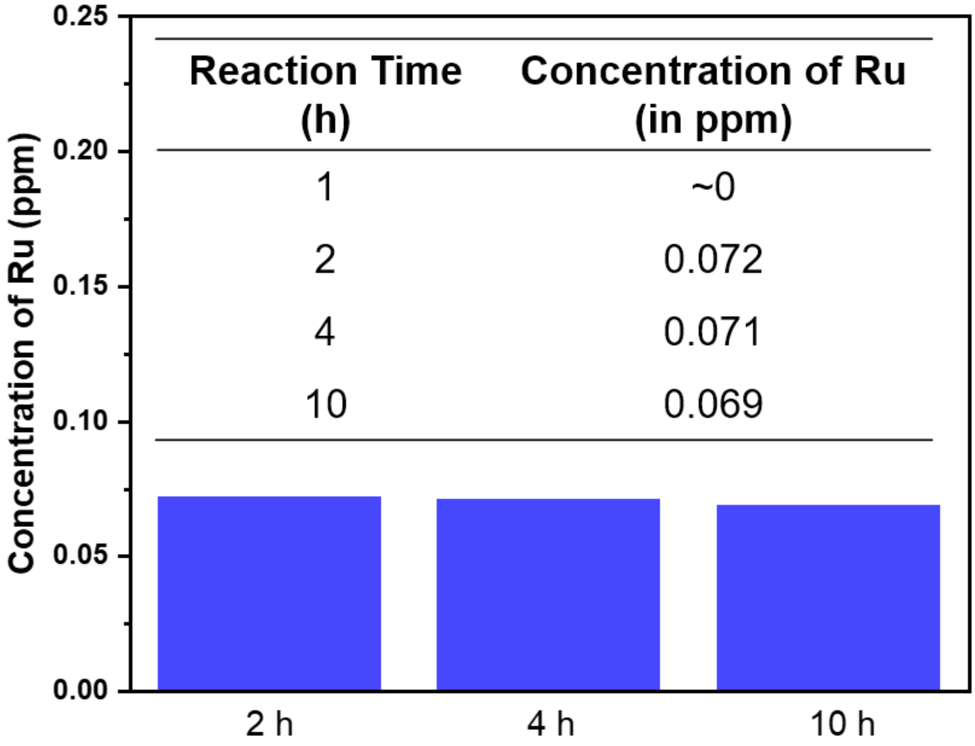

Supplementary Fig. 31. ICP-OES data of Ru species in electrolyte after different reaction time. Ru-$NiPS_3$ dipped into the electrolyte for 16 h was chosen as the sample

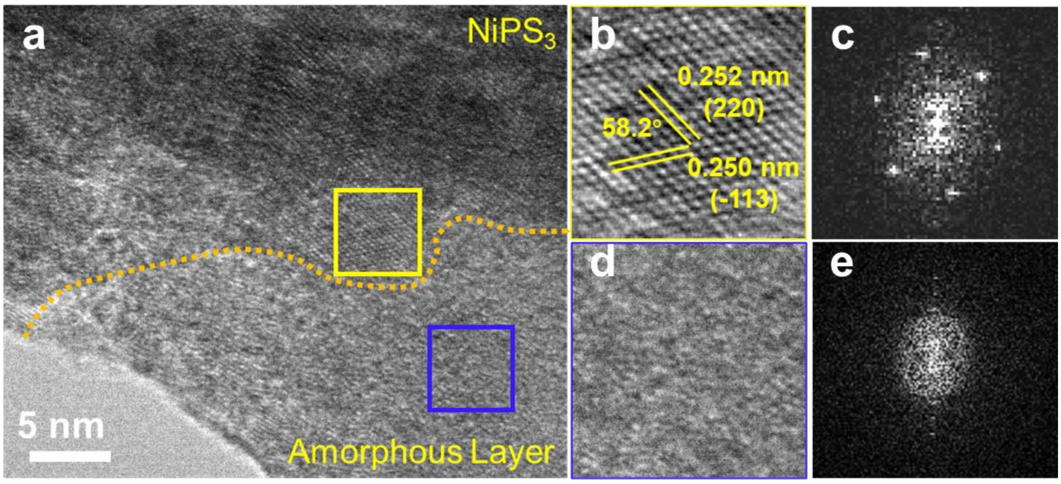

Supplementary Fig. 32. (a) HRTEM image of $NiPS_3$ NSs after HER stability test. (b) and (c) was the enlarged image of the crystalline part in (a) (yellow square) and the corresponding FFT diffractogram, respectively. (d) and (e) was the enlarged image of the amorphous layer in (a) (blue square) and the corresponding FFT diffractogram.

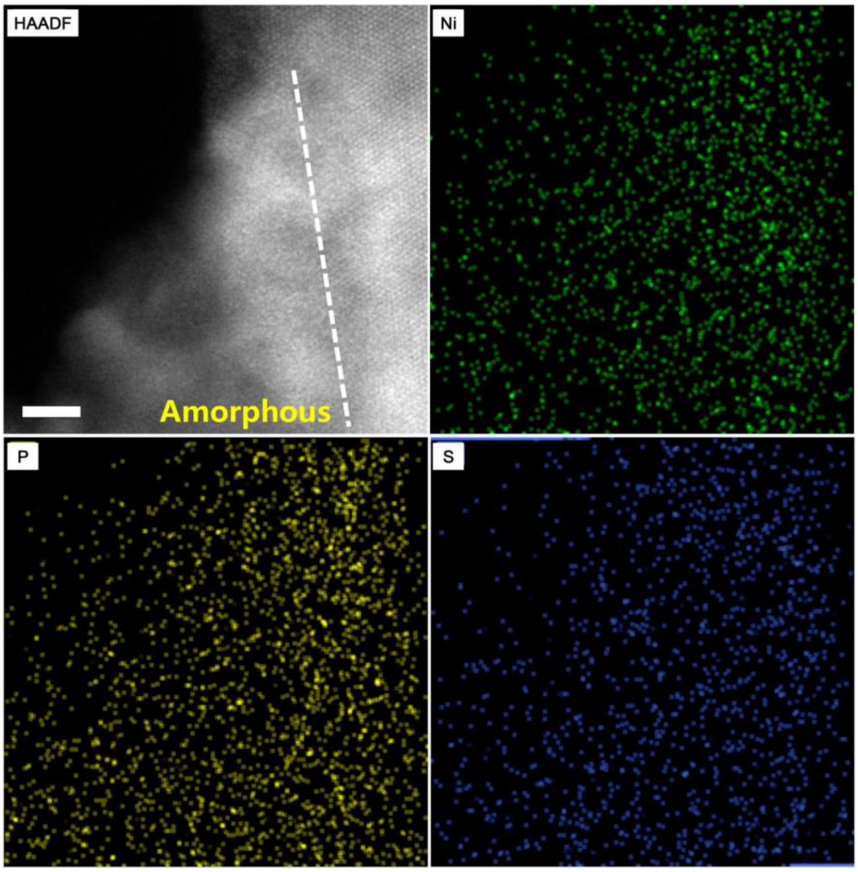

Supplementary Fig. 33. AC-HAADF stem image of $NiPS_3$ NSs after HER test and the corresponding EDS elemental mapping for Ni, P, and S (scale bar 1 nm).

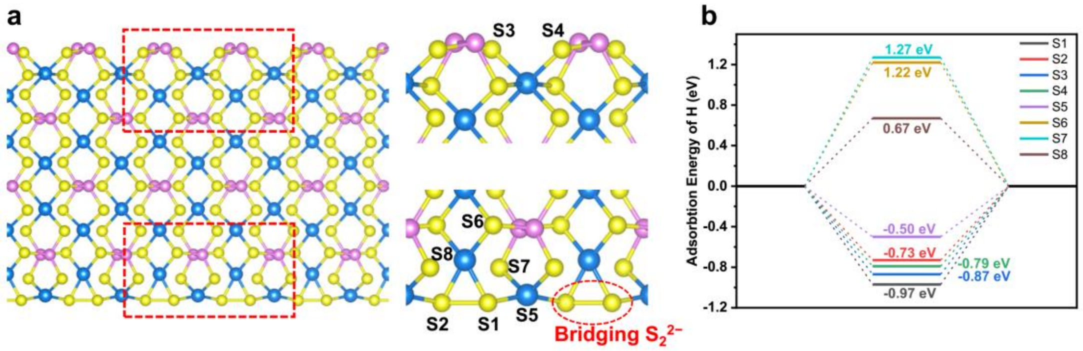

Supplementary Fig. 34. (a) Atomic model of $NiPS_3$ with edge bridging S_2^{2-} species and eight possible active sites. (b) the corresponding hydrogen adsorption energy for the eight potential active sites.

Supplementary Table 1 ICP-OES results of $NiPS_3$ and Ru-$NiPS_3$ electrodes

Sample	Element	Content (wt.%)
**$NiPS_3$**	Ni	6.80
	P	4.62
	S	10.60
**Ru-$NiPS_3$**	Ni	6.24
	P	4.99
	S	11.74
	Ru	0.81

Supplementary Table 2 The ICP-OES results for other $NiPS_3$ electrodes treated with $RuCl_3$ solution for varying durations

**Dipping Duration**	**Element**	**Content (wt. %)**
**0.5 h**	Ni	4.59
	P	5.00
	S	11.61
	Ru	0.16
**2 h**	Ni	4.31
	P	5.22
	S	12.68
	Ru	0.22
**4 h**	Ni	3.29
	P	4.70
	S	14.01
	Ru	0.36
**20 h**	Ni	5.77
	P	3.77
	S	11.70
	Ru	0.79

Supplementary Table 3 HER performances of each sample

Sample	η_{10} (mV)	Tafel Slope (mV dec^{-1})	j_0 (μA cm^{-2})	R_s (Ω)	R_{ct} (Ω)
**$NiPS_3$ powder**	266	115.1	48	3.1	23.1
**$NiPS_3$ NSs**	146	77.8	130	3.5	11.8
**Ru-$NiPS_3$ NSs**	58	64.0	1180	2.8	2.1

Supplementary Table 4 Comparisons of the catalytic performances of Ru-$NiPS_3$ in this work with other reported Ni-based electrocatalysts in 1 M KOH.

Sample	Overpotential (mV@10 mA cm^{-2})	Tafel Slope (mV dec^{-1})	Ref.
**Ru-$NiPS_3$**	**58**	**64.0**	**This work**
LSTL $NiPS_3$	158	95	[1]
$NiPS_3$/Ni_2P	85	82	[2]
$NiPS_3$/NF	74	86	[3]
mosaic $CoNiPS_3$/C nanosheets	140	60	[4]
$Co_{0.6}(VMnNiZn)_{0.4}PS_3$ nanosheets	65.9	65.5	[5]
V-$NiPS_3$	124	65.4	[6]
$Ni_{0.9}Fe_{0.1}PS_3$	72	73	[7]
$NiPS_3$@G	126	64	[8]
$Ni_{0.95}Co_{0.05}PS_3$	71	77	[9]
$NiPS_3$ −3.0 V + P	205	74	[10]
NF/Ni_3S_2@TiO_2	190	102.3	[11]
$Ni(OH)_2$/MoS_2	227	105	[12]
$Fe_5Ni_4S_8$/ FeNi foam	236	61.8	[13]
Ru-NiFeP/NF	56	68	[14]
Ni_5P_4-Ru/CC	54	52	[15]
$NiRu_2$@NC	53	38	[16]

Supplementary Table 5 Adsorption energy of different Ru doping sites

	Model	ΔE_{ads} (eV)
**$NiPS_3$-ac-Ru-1**	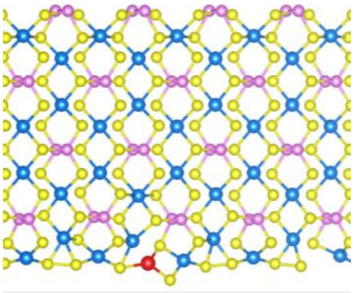	-7.8872
**$NiPS_3$-ac-Ru-2**	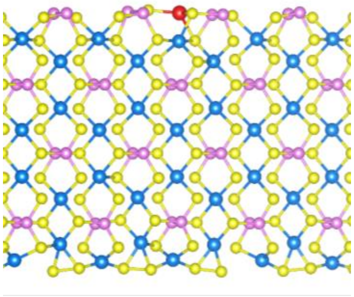	-7.41682
**$NiPS_3$-ac-Ru-3**	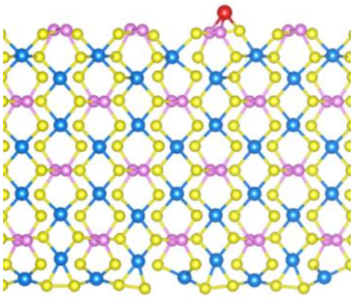	-6.6175

Note: *A negative value of ΔE_{ads} corresponds to an exothermic process, and a more negative value indicates a more stable Ru atom in $NiPS_3$ substrate, which is the indicator for a more stable calculation model. For $NiPS_3$-ac-Ru-1, the Ru atom was doped into the S_2^{2-} group, which connects with S atoms. As a comparison, the Ru atom in $NiPS_3$-ac-Ru-2 was doped into the lattice where there is no S_2^{2-} group around it. For $NiPS_3$-ac-Ru-3 Ru atom was adsorbed at the edge of $NiPS_3$.*

References

1. Li, X. et al. High-Yield Electrochemical production of large-sized and thinly layered $NiPS_3$ flakes for overall water splitting. *Small* **15**, 1902427 (2019).
2. Liang, Q. et al. Interfacing epitaxial dinickel phosphide to 2D nickel thiophosphate nanosheets for boosting electrocatalytic water splitting. *ACS Nano* **13**, 7975-7984 (2019).
3. Fang, L. et al. *In situ* formation of highly exposed $NiPS_3$ nanosheets on nickel foam as an efficient 3D electrocatalyst for overall water splitting. *Sustain. Energy Fuels* **5**, 2537-2544 (2021).
4. Liang, Q. et al. Mosaic-structured cobalt nickel thiophosphate nanosheets incorporated N-doped carbon for efficient and stable electrocatalytic water splitting. *Adv. Funct. Mater.* **28**, 1805075 (2018).
5. Wang, R. et al. Two-dimensional high-entropy metal phosphorus trichalcogenides for enhanced hydrogen evolution reaction. *ACS Nano* **16**, 3593-3603 (2022).
6. Tong, Y., Chen, P., Chen, L. & Cui, X. Dual vacancies confined in nickel phosphosulfide nanosheets enabling robust overall water splitting. *ChemSusChem* **14**, 2576-2584 (2021).
7. Song, B. et al. Tuning mixed nickel iron phosphosulfide nanosheet electrocatalysts for enhanced hydrogen and oxygen evolution. *ACS Catal.* **7**, 8549-8557 (2017).
8. Zhang, J., Cui, R., Li, X. a., Liu, X. & Huang, W. A nanohybrid consisting of $NiPS_3$ nanoparticles coupled with defective graphene as a pH-universal electrocatalyst for efficient hydrogen evolution. *J. Mater. Chem. A* **5**, 23536-23542 (2017).
9. Li, K., Rakov, D., Zhang, W. & Xu, P. Improving the intrinsic electrocatalytic hydrogen evolution activity of few-layer $NiPS_3$ by cobalt doping. *Chem. Commun.* **53**, 8199-8202 (2017).
10. Luxa, J., Cintl, S., Spejchalova, L., Lin, J.-Y. & Sofer, Z. Potential dependent electrochemical exfoliation of $NiPS_3$ and implications for hydrogen evolution reaction. *ACS Appl. Energy Mater.* **3**, 11992-11999 (2020).
11. Guo, D., Wan, Z., Fang, G., Zhu, M. & Xi, B. A tandem interfaced (Ni_3S_2-MoS_2)@ TiO_2

composite fabricated by atomic layer deposition as efficient HER electrocatalyst. *Small* **18**, 2201896 (2022).

12. Zhao, G. et al. Epitaxial growth of $Ni(OH)_2$ nanoclusters on MoS_2 nanosheets for enhanced alkaline hydrogen evolution reaction. *Nanoscale* **10**, 19074-19081 (2018).
13. Wu, Y. et al. Coupling interface constructions of $MoS_2/Fe_5Ni_4S_8$ heterostructures for efficient electrochemical water splitting. *Adv. Mater.* **30**, 1803151 (2018).
14. Lin, Y. et al. Ru doped bimetallic phosphide derived from 2D metal organic framework as active and robust electrocatalyst for water splitting. *Appl. Surf. Sci.* **536**, 147952 (2021).
15. He, Q. et al. Achieving efficient alkaline hydrogen evolution reaction over a Ni_5P_4 catalyst incorporating single-atomic Ru sites. *Adv. Mater.* **32**, 1906972 (2020).
16. Xu, S. et al. NiRu nanoparticles encapsulated in a nitrogen-doped carbon matrix as a highly efficient electrocatalyst for the hydrogen evolution reaction. *Dalton Trans.* **49**, 13647-13654 (2020).
