# Supplementary material for: Unraveling and leveraging in situ surface amorphization for enhanced hydrogen evolution reaction in alkaline media
Source: Nat Commun. 2023 Oct 13;14:6462. doi: 10.1038/s41467-023-42221-6 (PMC10575887; doi:10.1038/s41467-023-42221-6)
Supplement: Supplementary file 3 — Description of Additional Supplementary Files [file 41467_2023_42221_MOESM3_ESM.pdf]

Description of Additional Supplementary Files

File Name: Supplementary Movie 1
Description: *In situ* liquid TEM measurement of Ru-$NiPS_3$ NSs in alkaline without adding external applied voltage. There was no significant morphological change observed after electron beam irradiation.

File Name: Supplementary Movie 2
Description: *In situ* liquid TEM measurement of Ru-$NiPS_3$ NSs in alkaline under a constant current of -5 nA vs. Pt. Under those conditions, we observed significant morphological structural evolution.

File Name: Supplementary Movie 3
Description: *In situ* liquid TEM measurement of Ru-$NiPS_3$ NSs after long-term stability test. It was found that after a period of testing, the morphology is not expected to undergo any further significant changes.
